# Exosomes: Potential Disease Biomarkers and New Therapeutic Targets

**DOI:** 10.3390/biomedicines9081061

**Published:** 2021-08-20

**Authors:** Maria I. Mosquera-Heredia, Luis C. Morales, Oscar M. Vidal, Ernesto Barceló, Carlos Silvera-Redondo, Jorge I. Vélez, Pilar Garavito-Galofre

**Affiliations:** 1 Department of Medicine, Universidad del Norte, Barranquilla 081007, Colombia; burbanoc@uninorte.edu.co (L.C.M.); oorjuela@uninorte.edu.co (O.M.V.); csilvera@uninorte.edu.co (C.S.-R.); 2 Instituto Colombiano de Neuropedagogía, Barranquilla 080020, Colombia; erbarcelo@yahoo.com; 3 Department of Industrial Engineering, Universidad del Norte, Barranquilla 081007, Colombia; jvelezv@uninorte.edu.co

**Keywords:** exosome, biogenesis, intercellular communication, biomarker, therapeutic vehicle

## Abstract

Exosomes are extracellular vesicles released by cells, both constitutively and after cell activation, and are present in different types of biological fluid. Exosomes are involved in the pathogenesis of diseases, such as cancer, neurodegenerative diseases, pregnancy disorders and cardiovascular diseases, and have emerged as potential non-invasive biomarkers for the detection, prognosis and therapeutics of a myriad of diseases. In this review, we describe recent advances related to the regulatory mechanisms of exosome biogenesis, release and molecular composition, as well as their role in health and disease, and their potential use as disease biomarkers and therapeutic targets. In addition, the advantages and disadvantages of their main isolation methods, characterization and cargo analysis, as well as the experimental methods used for exosome-mediated drug delivery, are discussed. Finally, we present potential perspectives for the use of exosomes in future clinical practice.

## 1. Introduction

Exosomes are a type of extracellular vesicles involved in a cell–cell communication, a fundamental process in living organisms to regulate their metabolism and promote their adaptation and survival [1].

These vesicles have a spherical shape, delimited by a lipid membrane, with an average diameter of 30–150 nm [2]. Exosomes are known to contain various bioactive molecules, including proteins, nucleic acids, lipids and metabolites. It has been shown that exosomes are released by healthy and diseased cells, both constitutively and after cell activation, into the extracellular space, following the fusion of late endosomes and multivesicular bodies (MVBs) with the plasma membrane [1].

Although exosomes were first observed in the 1980s [3,4], it wasn’t until the early 1990s, when it was proposed that exosomes can transport signaling molecules, to adjacent and distant cells, regulating the physiology of recipient cells and participating in the onset and progression of multiple diseases [5]. Therefore, there is a growing interest in exploring exosome’s potential role as non-invasive biomarkers for the early detection and prognosis of diseases, as they are present in different types of biological fluids, such as blood, breast milk, urine, saliva, seminal fluid, amniotic fluid, cerebrospinal fluid and synovial fluid, among others [6]. Additionally, due to their biocompatibility, low immunogenicity and ability to cross biological barriers, they have also been proposed as drug delivery vehicles in tissue-specific therapies [7].

Different techniques, including proteomic [8] and related micro-methodologies [9], lipidomics [10] and transcriptomics techniques [11] have been designed for the isolation, characterization and analysis of biomarker constituents, which are important for assessing disease progression. Also, exosomal cargo has been processed using these methods. As expected, each of these methods has its own advantages and limitations, which makes challenging their selection. Methods for drug delivery through exosomes have also been proposed, which include loading of drugs into these nanovesicles, as well as the release and distribution of them in specific tissues. In this review, we explore the role of exosomes in health and disease, analyze the advantages and limitations of the techniques designed for their study, and discuss the prospects of their potential clinical applications as biomarkers and new therapeutic agents.

## 2. Exosome Biogenesis, Release and Intercellular Communication

**Figure 1:**
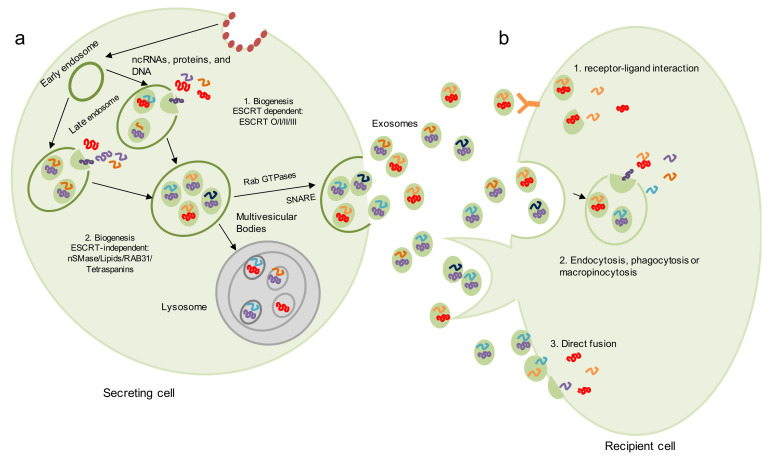
Biogenesis of exosomes and cell-to-cell communication. (**a**) Exosome biogenesis starts with the inward budding of the plasma membrane and the participation of proteins, such as clathrin. Endocytosis results in the formation of an early endosome, which encapsulates cellular proteins and genetic material present in the cytoplasm, becoming a late endosome. Then, multivesicular bodies (MVBs) containing exosomes are formed. The generation of exosomes can occur by two main pathways: (1) a ESCRT-dependent pathway and (2) a ESCRT-independent pathway. MVBs can be directed to the lysosome for degradation and recycling of MVB components or to the plasma membrane, and exosomes are released into the extracellular space by Rab small GTPases or R-SNARE. (**b**) Secreted exosomes can be taken up by recipient cells through several mechanisms, including (1) receptor-ligand interaction, (2) exosomal internalization by endocytosis, phagocytosis or micropinocytosis and (3) direct membrane fusion.

Exosomal biogenesis is initiated by the activation of a receptor located on the surface of the plasma membrane [12]. The stimulation of this receptor initiates the endocytosis of the ligand-receptor complex with the internal membrane budding and the participation of a particular set of proteins, such as clathrin [13,14]. Endocytosis results in the formation of an early endosome, which encapsulates cellular proteins and genetic material (mRNA, non-coding RNA and DNA) present in the cytoplasm [2,15]. Then, the early endosome matures and becomes a late endosome [13]; which contains smaller vesicles or intraluminal vesicles (ILVs), also known as the multivesicular body (MVB) (Figure 1a) [13].

A set of proteins involved in exosome formation are the components of the endosomal sorting complexes required for transport (ESCRT) machinery. This is a set of cytosolic proteins, induced by ubiquitination signaling and internalized in early endosomes, allowing properly ubiquitinated proteins to be loaded into the MVBs. The localization of proteins within exosomes does not occur randomly; instead, cells actively move proteins into the MVBs, depending on their labeling. It has been shown that proteins, ubiquitinated in a Lys63 branching pattern, are preferentially transferred into nascent exosomes, while proteins ubiquitinated in other lysine residues are usually degraded by the proteasome [16]. However, there are some exceptions, wherein non-ubiquitylated proteins can also be loaded into these vesicles [17].

ESCRT consists of four complexes, listed in order of action; namely, ESCRT-0, ESCRT-I, ESCRT-II and ESCRT-III. These complexes cooperate with specific molecules, such as VPS4 proteins (VPS4A, VPS4B, lysosomal trafficking regulator interacting protein 5 [LIP5]) and Bro1 complexes (ALIX, his-tyrosine phosphatase domain protein [HDPTP], BRO1 domain and CAAX motif-containing protein [BROX]) [18]. ESCRT-0 is activated by phosphatidylinositol 3-phosphate and ubiquitinated by molecules present on the outside of the endosomal membrane, and is responsible for recruiting ESCRT-I through the interaction between prosaposin domains (PSAP), a substrate of hepatocyte growth factor-regulated tyrosine kinase (HRS PSAP) and the TSG101 protein in ESCRT-I [1]. The ESCRT-I complex is essential for cargo sorting at the MVB and, together with ESCRT-II, acts at the membrane to drive ILV budding. In addition, ESCRT-II regulates the formation of the ESCRT-III complex, which in turn is responsible for the sorting and concentration of MVB cargo, as well as for the bud splitting to form the ILVs [19]. Some studies indicate that there are ESCRT-independent mechanisms of ILV formation and exosome biogenesis [20,21,22,23]. One of these mechanisms depends on the enzyme neutral sphingomyelinase (nSMase), since cells in which the ESCRT machinery has been depleted continue to generate CD63-positive exosomes. nSMase hydrolyses sphingomyelin to ceramide, suggesting that ceramide has a crucial role in protein sorting into ILVs [20,21].

Tetraspanins, a specific class of membrane proteins, are demonstrated to be involved in another ESCRT-independent pathway of cargo selection and exosome formation [2]. This process involves the organization of the endosomal membrane into specialized domains, known as tetraspanins-enriched membrane domains (TEMs), which are proteins needed to facilitate vesicular fusion and/or fission [13]. Additionally, TEMs also recruit potential ligands for the receptor-mediated internalization of exosomes by the recipient cell. Several members of the tetraspanins family, including CD9, CD63 and CD81, are highly enriched in exosomal membranes and serve as marker proteins for the vesicles [14].

Recently, another ESCRT-independent exosome-biogenesis pathway, dependent on RAB31, has been described [24]. It was found that active RAB31 drives epidermal growth factor receptor (EGFR) entry into MVBs to form ILVs and exosomes, with the involvement of flotillin in lipid-raft microdomains. Furthermore, it was demonstrated that RAB31 recruits TBC1D2B to inactivate RAB7 and thereby suppresses the fusion of MVBs with lysosomes, thus promoting their release into the extracellular space [24].

Other ESCRT-independent mechanisms of ILV formation may involve phospholipase D2 (PLD2) and ADP GTPase ribosylation factor 6 (ARF6), or heat shock proteins [20]. Once formed, late endosomes are destined to fuse either with the lysosome, leading to degradation of the vesicle contents, or with the plasma membrane allowing exosomes to be released into the extracellular space [13]. 

Once MVBs are formed, two alternative destinations are possible: (1) to fuse with the lysosomes, so that their contents are degraded, or (2) to fuse with the plasma membrane to release their ILVs as exosomes [1,25]. The mechanisms that prevent lysosomal degradation in favor of exosome secretion provide a powerful control point for the regulation of the release of these vesicles into the extracellular medium, although they are not yet entirely understood [26]. It has been suggested, for example, that ESCRT-dependent and ESCRT-independent exosome formation seems to lead to lysosomal secretion and degradation of MVBs, respectively. Other molecules that appear to be involved in this process are TSG101 and tetraspanin 6. ISGylation (protein conjugation by ISG15) of TSG101 inhibits the secretion of exosomes, while mutations that alter this conjugation, can increase their secretion [27]. On the other hand, overexpression of tetraspanin 6 decreases the rate of lysosomal degradation of C-terminal fragments of amyloid precursor proteins and increases exosome secretion, probably through syntenin recruitment [28].

Once the fusion of MVBs with the plasma membrane is defined as their final destination, various mechanisms of exosomal secretion will be required. One of these mechanisms requires the YKT6 protein, which is one of the R-SNARE molecules involved in vesicular transport between secretory compartments [29]. With their participation, the cytoskeleton and the contractile machinery of the cell move, reducing the distance between the vesicle membrane and the plasma membrane. Gross et al. [29] demonstrated that YKT6 depletion decreased TSG101, WNT3A and VPS26/35 levels in exosomes secreted by human embryonic kidney HEK293 cells [29], and Ruiz-Martínez et al. [30] observed reduced TSG101 levels associated to exosomes after YKT6 depletion in human lung cancer A549 cells [30].

Rab proteins have also been reported to play a role in exosome secretion. The first to be associated with this process was Rab11, as overexpression of a dominant negative mutant Rab11 protein was shown to decrease exosome release from K562 cells [31]. Hsu et al. [32] showed that a decrease in Rab35 is directly proportional to the release of the proteolipid protein associated with exosomes of Oli-neu cells, possibly due to reduced coupling of MVBs to the plasma membrane [32]. This result was later confirmed using the same model [33]. Rab27A and Rab27B have also been shown to have important roles in the spontaneous secretion of MHC-II in exosomes released by HeLa cells [34]. Other RAB-GTPases, which are involved in several processes related to vesicle transport within cells, are RAB2B, RAB4, RAB5A, RAB7 and RAB9A [18].

Once secreted, exosomes need to interact with a receptor cell and induce changes in it in order to fulfill their role as messengers in intercellular communication processes. Three main mechanisms for exosome-mediated intercellular communication have been proposed so far [35] (Figure 1b). The first mechanism consists of the exosomal internalization by phagocytosis, micropinocytosis and micropinocytosis or endocytosis, a process by which the contents of the exosome, as well as its membrane, are engulfed by a target cell within a newly formed vesicle known as a phagosome [36]. Endocytosis can be mediated by clathrin [37], lipid rafts [38] or be dependent on heparin sulfate proteoglycans [39]. Endocytosis appears to be the main method of entry for extracellular vesicles. However, clathrin-mediated endocytosis is one of the canonical pathways of exosome uptake [40]. Evidence shows that endocytosis is usually a temperature-influenced rapid process, with exosomes being internalizing into recipient cells as early as 15 min after initial contact [41]. Furthermore, it has been reported that when cells are incubated at 4 °C, their ability to internalize exosomes is drastically reduced, suggesting that uptake is an energy-intensive process [42].

For phagocytosis to occur, the participation of enzymes, such as phosphatidylinositol-3-kinase and phospholipase C is necessary [40]. This pathway of exosome internalization is predominantly used by immune cells, such as macrophages and dendritic cells, which depend on PI3K and the activity of the actin cytoskeleton [43].

Macropinocytosis, on the other hand, uses actin-driven lamellipodia to induce the invagination of the plasma membrane to form intracellular compartments, called pinosomes, for the nonspecific uptake of soluble extracellular molecules, nutrients and antigens [44]. This macropinosome matures and then fuses with the lysosome for degradation or with the plasma membrane for recycling. This process is highly dependent on growth factors and is regulated by cholesterol, recruitment of the GTPase protein Rac1, the Na+/H+ exchanger and, in some cases, by dynamin [44]. Pinocytosis is one of the least-utilized exosomal internalization pathways [40].

Another mechanism for exosome-mediated intercellular communication consists in the interaction of exosomal membrane proteins with receptors present on the target cells that activate the corresponding intracellular signaling. Several classical ligand/receptor pairs have been described in these interactions, each of which is probably specific to a given exosomal cell source and receptor cell type. An example of this mechanism is the antigen associated with lymphocyte function 1 (LFA-1), located on the surface of activated T cells or antigen-presenting cells, which acts as a receptor by binding to ICAM-1. This latter protein is present on the membranes of exosomes released by mature dendritic cells [45,46]. Interaction between proteins that are cleaved from the exosomal membrane and their corresponding receptors on the target cells have been also shown to occur. This is the case of the tumor necrosis factor receptor 1 (TNFR1) on vascular endothelial cells [47] or the CD46 receptor of ovarian adenocarcinoma cell lines [48].

Finally, another commonly observed mechanism is the fusion between the plasma membrane of a recipient cell and the consequent transfer of its contents favored by an acidic pH, perhaps due to differences in lipid content or the overall ionic charge of the exosome surface after release [40]. This mechanism has been shown to be important in the transfer of nucleic acids, proteins and exosomal lipids [49,50].

## 3. Exosomal Molecular Contents

Exosomes are vesicles with a lipid bilayer that carry RNA and proteins, and possibly other molecules of biological importance. According to the latest version of the exosome content database, ExoCarta (http://www.exocarta.org/ accessed on 9 August 2021), 41,860 proteins, 4946 RNAs and 1116 lipids have been identified in exosomes released by cells from 10 different species in 286 studies [51].

### 3.1. Proteins

The study of the proteins contained within the exosomes indicate that the origin of these vesicles is from living cells and not from apoptotic cells [52], and that some of them are common and exclusive to all exosomes, while others are highly dependent on the cell type of origin. Common proteins in exosomes include tetraspanins (CD9, CD63, CD81 and CD82), proteins needed for transport and their ability to fuse with other cell membranes (annexins, Rab proteins, flotillin), proteins associated with MVB biogenesis (Alix, TSG101, YWHAE, ubiquitin, LAMP-2B), and heat shock proteins (Hsc70, Hsp90) [40,53]. These vesicles also carry a variety of cytoskeletal proteins (i.e., actin, tubulin, prophylaxis, cofilin, sinenin, moesin, albumin) and metabolic enzymes, such as glyceraldehyde-3-phosphate dehydrogenase, pyruvate kinase, ATPase and fatty acid synthase (Table 1) [26,40]. Some metabolites, including carboxylic acids, carnitines, biogenic amines, vitamins and cyclic alcohols, have also been identified within exosomes [54].

Some examples of cell-dependent exosomal proteins include major histocompatibility complex (MHC) II and CD86, which are contained in exosomes released by antigen-presenting cells, and MFG-E8/lactadherin, which are contained in exosomes from immature dendritic cells. On the other hand, cardiomyocyte exosomes contain HP60; those from platelets contain P-selectin and granzymes, while exosomes from cytotoxic T cells, reticulocytes and enterocytes carry von Willebrand factor and perforin, α4β1 and immunoglobulin A33, respectively [6,55].

### 3.2. RNA

In addition to protein loading, exosomes also carry RNA, including microRNA (miRNA), non-coding RNA (ncRNA), mitochondrial RNA (mtRNA) and messenger RNA (mRNA) (see Table 1) [57,58]. RNA is considered the main functional component of the exosome; once in the receptor cell, RNA plays the same role as in the cell of origin [59]. It has been shown that the RNA load reflects the state and cytoplasmic content of the cell of origin. Thus, exosomes provide a method for the exchange of genetic information between cells [60]. Research suggests that exosomal miRNAs are functional, in the recipient cell, by suppressing the expression of its target genes [61,62,63]. The ability of these miRNAs to exert a biological effect indicates that they are stable as long as they remain associated with the cellular proteins that make them functional. In turn, mRNA transcripts can be easily translated into proteins in the cell receiving the vesicles. Valadi et al. [60] were among the first to demonstrate that exosomal mRNA from mouse cells could be translated into murine proteins in human mast cells. However, most of these transcripts appear to be highly enriched in the untranslated 3′ regions, suggesting that the mRNA contained in these vesicles may play a regulatory, rather than a functional, role [64].

Overall, different RNAs in extracellular vesicles have been classified in (1) functional RNAs, such as mRNAs and miRNAs; (2) RNAs potentially functional but without an established intercellular communication, namely piRNAs and vtRNAs and (3) fragments of RNAs such us tRNA, mRNAs and rRNAs that can be either functional or non-functional [65]. Thus, the delivery of mRNA-encoding wild-type proteins has been proposed as an attractive approach in the treatment of some inherited genetic diseases. Indeed, MSC-derived microvesicles were shown to release exosomes that transfer functional cystinosin and its mRNA, increasing the amount of the protein in skin fibroblasts from patients with cystinosis, subsequently reducing cysteine accumulation in these cells in vitro [66].

Similar to cellular RNAs, virus-specific-encoded RNAs can also be packaged into the exosomes of infected cells, and infect the recipient cell [67]. Indeed, it has been postulated that the human immunodeficiency virus’ (HIV) envelope is in fact an exosome that carries viral RNA [68]. Moreover, it has been shown that the Epstein–Barr virus encodes BHRF1 and BART viral miRNAs that can be eliminated in the nanovesicles of infected B cells and suppress the immunoregulatory *CXCL11* gene in uninfected dendritic cell-derived monocytes [69]. Thus, it is clear that viruses have developed strategies to seize the host’s exosomal machinery in order to propagate and mitigate their antiviral response.

### 3.3. Lipids

Unlike RNA and proteins, the lipid composition of exosomes has been less studied, and hence is a relatively uninformed area. Exosomes are known to be mainly enriched with sphingolipids, cholesterol, phosphatidylserine, phosphatidylinositol-3-phosphate and bis-monoacylglycero-phosphate (BMP) [70]. It has been shown that the lipid composition of ILVs appears to vary by the stage of maturity of MVBs, with ILVs being especially enriched with cholesterol in the early stages, and with BMP in later stages [40,56,71].

Recently, Peterka et al. [72] compared the lipid composition of circulating exosomes with the lipids present in the plasma of 12 healthy volunteers, using three mass spectrometry approaches to identify important differences. The main finding of this study was a significant increase in triglycerides, followed by diacylglycerol in the exosomes, compared with that found in the plasma, while phosphatidylethanolamine was only detected in these vesicles. 

Lipids found in the exosome membrane confirm that they have a different origin than microvesicles, which are derived by direct sprouting of the plasma membrane. In this sense, researchers reported a lipid composition consisting of a lower amount of lysophosphatidylcholine and phosphatidylcholine and a higher amount of sphingomyelin and ceramide, with respect to the concentrations present in the plasma membrane (Table 1). This lipid composition prolongs the halflife of exosomes in plasma, since this may be more resistant to degradation [40,56,72]. Future research will help increase knowledge about exosomal lipids and better understand the biology of these vesicles, facilitating their use in the clinical setting.

## 4. Role of Exosomes in Health

The functional relevance of exosomes, released into circulation, lies in the effect that these nanovesicles have in delivering their contents to recipient cells, participating in physiological processes, such as cell proliferation, immune response, lactation and neuronal and cardiovascular function [73].

### 4.1. Immunomodulation

Regarding their role in immunomodulation, mature dendritic cells (DCs) are known to release exosomes with surface markers, such as the MHC class I and II, and therefore can stimulate CD8 and CD4 T cells, which induces the adaptive immune response [74]. At relatively high concentrations, exosomes derived from antigen-presenting cells (APCs) that carry peptide-MHC complexes (p-MHC) function as Ag-presenting vesicles for T-cell clones, lines and hybrids, and for active T-cells [45].

When infections occur, DCs absorb antigens and MHC molecules, which activate auxiliary T cells. Subsequently, T cells activate B cells and lead to an increase in the production and release of exosomes, which also contain MHCs. These B cell-derived exosomes then stimulate CD4 [6]. The strength of the activation effect seems to depend on the physiological state of the cell, as mature DCs will release exosomes more efficiently for the induction of T-cell activation than immature DCs [46]. In this sense, exosomes derived from immature DCs need to be processed by antigen-presenting cells before they can induce T-cell activation. The inability of such exosomes to directly activate T cells could be a consequence of insufficient amounts of T cell activating molecules (i.e., CD40, CD86 and ICAM-1) [75]. In addition, exosomes released by immature DCs have been proven to induce T cells apoptosis by promoting a tolerant immune response. These exosomes can also balance pro-inflammatory and anti-inflammatory effector T cells by inducing auxiliary T cells to differentiate into regulatory T cells [73], which could be achieved by the action exerted by exosome-transported IL-10, thus inducing a tolerance response [76].

### 4.2. Feto-Maternal Interface

During pregnancy, the intimate contact of fetal cells and maternal cells represents a substantial immune challenge. Inflammation, for example, is necessary for tissue remodeling and effective implantation, although it must be regulated in order to avoid miscarriage and other pregnancy complications. After implantation, a shift is made to a less inflammatory environment to allow for fetal development. Finally, at the end of the third trimester, close to delivery, a proinflammatory environment becomes predominant again [77]. Thus, an active participation of exosomes released at the maternal-fetal interface has been proposed for the establishment of an immunoprivileged environment for fetal development [78].

Exosomes, derived from human endometrial epithelial cells, were shown to be taken up by trophoblasts to enhance their adhesive potential, in part through enhanced focal adhesion kinase signaling [79], and some miRNAs, such as miR-30d, which induces the overexpression of genes involved in embryonic adhesion, such as integrin beta-3, integrin alpha-7 and cadherin-5 [80]. On the other hand, miR-520c-3p modulates chorionic villous trophoblast invasion mediated by CD44 [81]. Regarding the proinflammatory state required at this stage, it is known that trophoblast-derived exosomes can regulate the recruitment and differentiation of monocytes into tissue macrophages by inducing them to secrete the cytokines and chemokines necessary for trophoblast growth and survival [82]. Likewise, macrophages internalized by placental cells increased the release of proinflammatory cytokines, such as IL-6, IL-8 and IL-10 [83].

Interestingly, thanks to the immunosuppressive character of placenta-derived vesicles, which is partially mediated by the expression of the proapoptotic molecules FasL (Fas ligand), PD-L1 and TRAIL, this condition does not directly affect the embryo. In addition, trophoblast-derived exosomes have been shown to also induce T cell differentiation into Treg (regulatory T) cells mediated by HSPE1 (heat shock protein 10kDa 1) [84]. These exosomes also express a particular combination of four MHC class I molecules: the classical HLA-C molecules, and the non-classical class I molecules HLA-E, HLA-F and HLA-G. The expression of HLA-E and G allows them to evade NK cell-mediated cytotoxicity contributing to the immunotolerance required for successful pregnancy [78]. 

### 4.3. Mother-To-Baby Network

Exosomes are also known to be involved in maintaining the health of infants, being their main function to transport proteins and RNA in the mother’s milk to the infant’s intestinal system, protecting them from acidity and degradation, thus allowing them to remain intact and to be absorbed [85,86]. These molecules significantly promote the viability of intestinal and gingival epithelial cells, increasing their proliferation and stimulating their activity [87]. For example, some exosomal circular RNAs (circRNAs), found in colostrum, play important biological roles through binding to their respective miRNAs, which promote vascular endothelial growth factor expression and induce proliferation and migration of small intestinal epithelial cells. Breast milk exosomes have also been shown to directly enhance gingival epithelial cell migration through p38 mitogen-activated protein kinases and cytoskeleton remodeling [76]. It has been hypothesized that breast milk extracellular vesicles are involved in the selective inhibition of toll-like receptors 3, 4 and 9 (TLR3, TLR4 and TLR9) through the transport of epidermal growth factor, and, probably, cystatin-B cathepsin inhibitor (CSTB) [76]. These proteins inhibit the signaling of these receptors, thus protecting the intestinal epithelium against apoptosis and favoring tolerance to the microbiota. 

### 4.4. Exosomes in the Nervous System

Brain exosomes also play an important role in central nervous system (CNS) homeostasis and can contribute to the communication between cells whether they are neighboring or distant [6]. Oligodendrocyte-derived exosomes contain myelin proteins and lipids, including Folch-Lees proteolipid, CNPase, myelin-associated glycoprotein, oligodendrocyte myelin glycoprotein, cholesterol, and sphingolipids, as well as other molecules, such as heat shock proteins, which act directly on other oligodendrocytes, neurons or microglia to regulate myelin biogenesis, degradation and maintenance, as well as to provide neuronal trophic support [88]. Additionally, oligodendroglial exosomes exert beneficial effects on neurons, potentially through the transfer of protective proteins, such as catalase and superoxide dismutase. In this sense, neurons that take up these exosomes exhibit increased tolerance to oxidative stress or starvation [89]. On the other hand, following glutamatergic stimulation, cortical neurons secrete exosomes containing neuronal cell adhesion molecule L1, as well as subunits of the alpha-amino-3-hydroxy-5-methyl-4-isoxazolepropionate receptor GluR2 and GluR3, suggesting a role for exosomes in synaptic plasticity [90].

In the peripheral nervous system, Schwann cells secrete exosomes that are internalized by neighboring axons, providing local axonal support and enhancing axonal regeneration after nerve damage [91]. Gomez-Molina et al. provide evidence of two-way communication through exosomes from the brain to the rest of the body [92]. However, the mechanism used by these nanovesicles to cross the blood-brain barrier (BBB) is not yet completely understood. It has been proposed that exosomes cross the BBB by endocytosis or transcytosis, mediated by adsorption by brain macrovascular endothelial cells [93]. Chen et al. showed that endocytosis could be dependent on clathrin and/or caveolae. Once exosomes are internalized, they accumulate in the endothelial cell endosomes with the consequent formation of MVBs and their subsequent release into the bloodstream [94], where it is easier to detect them. Therefore, molecules that are part of their cargo are potential non-invasive biomarkers of CNS diseases [95].

### 4.5. Exosomes in the Cardiovascular System

The cardiovascular system also relies heavily on exosomes for optimal functioning. Recently, a wide variety of biomolecules have been identified in exosomes secreted by cardiomyocytes in several studies [96]. For instance, some important proteins, such as Hsp20, which promotes angiogenesis, and just as Hsp70, improves cardiac function. On the other hand, Hsp60 improves immune response and TNF-α contributes to cardiac remodeling [96]. Exosomes released by cardiomyocytes are also capable of transferring glucose transporters (GLUTs) into the cells that make up the coronary microvascular endothelium, where these transferred GLUTs increase glucose uptake and metabolism. Maintaining this disposition is essential to achieve a metabolic coupling between both cell types, especially considering that the heart has no appreciable reserves of metabolic fuels and the supply of nutrients and oxygen must be continuous and regulated. In this sense, cardiomyocytes use exosomes to regulate glucose transport in their associated endothelial cell [97]. In addition, it has been shown that DNA and RNA can be transferred between different cell types to induce changes in gene expression within the recipient cells [96,98].

### 4.6. Exosomes, Stem Cells and Tissue Development

The best characterized stromal support function of mesenchymal stem cells (MSCs) is the maintenance of hematopoietic cell homeostasis. MSCs contribute to maintaining a microenvironmental niche that balances their quiescence, self-renewal and differentiation according to the body’s needs [99]. Likewise, given their ubiquitous tissue distribution, MSCs promote tissue homeostasis by responding to physiological changes or pathological processes [100].

It has been proposed that MSCs exert their effects not only through their differentiation potential, but also through their secreted product, consisting of soluble proteins and exosomes. The latter interact with cells in a paracrine and endocrine manner [101], and are readily endocytosed by cells in an injured tissue mainly due to the acidosis generated during injury. Of course, the ability to regenerate and maintain the homeostasis of the tissue microenvironment will largely depend on the biochemical potential of the protein and RNA cargo of these exosomes [102,103].

Exosomes secreted by MSCs have been shown to contain growth factors, such as transforming growth factor-β (TGF-β) and hepatic growth factor (HGF), and anti-inflammatory chemokines, such as IL-10 and IL-1 receptor antagonist (IL-1Ra), which are characteristic and efficient immunomodulatory molecules secreted in large quantities by MSCs. Additionally, MSCs contain indolamine 2,3-dioxygenase-1 and prostaglandin E2, antimicrobial peptides capable of destroying Gram-positive and Gram-negative bacterias [104]. These vesicles also carry a panel of miRNAs, including miR-21, miR-23a and miR-124, which are recognized to have immunosuppressive and anti-inflammatory functions [105]. Undoubtedly, the MSC secretome plays a role in reducing apoptosis and fibrosis; increasing angiogenesis and cell survival and differentiation [106].

## 5. Role of Exosomes in Diseases

Exosomes also play an important role in the pathogenesis of diseases, such as cancer, neurodegenerative diseases, cardiovascular diseases and pregnancy-related disorders, including preeclampsia and gestational diabetes mellitus [107].

### 5.1. Exosomes in Cancer

The role of these extracellular vesicles and their contents as possible contributors to oncogenesis, metastatic disease and resistance to chemotherapy is a rapidly expanding area of research in cancer biology. Exosomes have been implicated in processes, such as inflammation and infections, conditions that may promote an environment suitable for active proliferation and the accumulation of mutations that ultimately lead to the development of a malignant tumor [108]. Tumor-derived exosomes (TDEs), have the potential to contribute to cancer progression via paracrine signaling by developing heterogeneous satellite tumors close to the primary tumor and/or by triggering a more invasive phenotype [109]. TDEs also transform fibroblasts into cancer-associated fibroblasts (CAF) and induce tubulogenesis, which is a process that plays an important role in shaping the tumor microenvironment. Sung et al. reported that triple-negative breast cancer cells overexpressing integrin beta 4 (ITGB4) increased the level of this protein in cancer-associated fibroblasts through exosomal transfer. According to this study, ITGB4 induced lactate production and BCL2 Interacting Protein 3 Like (BNIP3L)-dependent mitophagy in CAFs. Consequently, proliferation, epithelial-to-mesenchymal transition and invasion of breast cancer cells were promoted [110]. Another study demonstrated that exosomal TGFβ in bladder cancer cells mediated the transformation of fibroblasts into CAFs, which promoted epithelial-to-mesenchymal transition, cell growth, migration and invasion of bladder cancer cells [111]. TDEs also participate in the development of metastasis by transporting factors into the bloodstream that will subsequently reach the premetastatic niche and facilitate its formation. Finally, TDEs have the ability to promote the escape of tumor cells from the action of the immune system, and to contribute to the development of therapy resistance [108].

Common causes of persistent inflammation leading to cancer development include hepatitis C or B virus infection leading to hepatocellular carcinoma, inflammatory bowel disease leading to colorectal cancer, Helicobacter pylori infection leading to gastric cancer, and human papillomavirus (HPV) infection leading to cervical cancer [112]. While inflammatory mediators (i.e., chemokines and cytokines) are the main players in initiating the inflammatory response, there is increasing evidence supporting the longer-term connection between inflammation and exosomes. For example, in inflammatory bowel disease, nanovesicles released by Polymorphonuclear neutrophils have been shown to carry proinflammatory miR-23a and miR-155, and myeloperoxidase leading to the generation of reactive oxygen species (ROS). Both these ROS and miRNA can induce genotoxic stress resulting in the formation of single and/or double strand breaks [113,114]. In gastritis and gastric cancer, serum exosomes from patients with chronic gastritis infected with *H. pylori* were shown to promote the expression of proinflammatory cytokines in gastric epithelial cells, thereby advancing inflammation [115]. Furthermore, in HPV infection and the development of cervical cancer, the deregulation of 17 different miRNAs, all of them inducers of inflammation, has been demonstrated in vitro [116]. All in all, the above are some examples of conditions that can promote a suitable environment for active proliferation and accumulation of mutations leading eventually to the development of a malignant tumor.

Through TDEs, cancer cells can create “self-like” cells with various mutational and functional configurations [117]. For instance, exosomal circRNA_100284 from arsenite-transformed liver cells has been shown to mediate malignant transformation of healthy L-02 liver cells by accelerating the cell cycle and promoting cell proliferation [118]. Likewise, both adriamycin (BCa)-resistant breast cancer cells and their exosomes induce M2 polarization of macrophages, which in turn, produce high levels of IL-10 to promote motility, proliferation, migration and invasion of BCa cells [119]. Regarding tumor progression through paracrine signaling to neighboring cells, some studies have shown that exosomes can be used as vehicles for this communication. For example, TDEs secreted by MIA PaCa-2 pancreatic cancer cells were found to carry Annexin A1, which influences the metastatic nature of the tumor by inducing epithelial-to-mesenchymal transition and increasing cell motility [120].

TDEs have the ability to transport immunomodulatory molecules, which is a crucial aspect of the antitumor immune response, recognized as a key feature of metastasis that can also contribute to resistance to therapy [121]. For example, breast cancer-derived exosomal programmed death-ligand 1 (PD-L1) was shown to bind to the PD-1 receptor on CD8 T cells, promoting their dysfunction, which in turn promotes tumor growth [122]. A recent review on the interaction between Natural killer (NK) cells and TDEs lists some studies demonstrating that these NK cells can interact with and capture PKH67-labeled exosomes derived from human tumor cells, including pancreatic cancer (L3. 6pl), myeloid leukemia (K562), T-cell leukemia (Jurkat), hepatoblastoma (HepG2), cervical cancer (HeLa), breast carcinoma (MCF-7) and multiple myeloma (SKO-007-subclone J3). This interaction succeeds in regulating NK cell function to the benefit of the tumor [123].

Studies have also demonstrated the involvement of some exosomal miRNAs and circRNAs in drug resistance. Indeed, circ_0032821, is overexpressed in exosomes of oxaliplatin (OXA)-resistant gastric cancer (GC) cells compared to OXA-sensitive GC cells. Zhong et al., concluded that circ_0032821 interacts with miR-515-5p, which in turns regulates the expression of the transcription factor SOX9, which could be involved in the chemoresistance of these cells [124]. Likewise, exosomal miRNA mediates tamoxifen resistance in breast cancer probably due to the on the *ADIPOQ* gene [125]. In prostate cancer (PCa), exosomal miR-27a could induce resistance to cisplatin, docetaxel and doxorubicin in recipient cells through degradation of p53 mRNA [126].

Exosomes have also been considered to participate in the efflux of chemotherapeutic agents from the tumor cell [127]. For example, in PCa, enzalutamide (Enz) resistant cells release significantly 2-3 times higher quantities of exosomes compared to the respective sensitive cells. It has also been shown that these resistant cells use exosomes to remove Enz from the cell and reduce the drug concentration [128]. Similarly, research suggests that B-cell lymphoma cells could eliminate doxorubicin and pixantrone through exosome secretion, and that inhibition of exosome biogenesis through indomethacin or genetic depletion of ABCA3 enhances the intracellular accumulation and cytostatic activity of both drugs both in vitro and in vivo experiments [129].

### 5.2. Exosomes in the Central Nervous System Diseases

Recent studies have shown that exosomes can also play key roles in neurodegenerative diseases, such as Alzheimer’s disease (AD) and Parkinson’s disease (PD) [130]. In AD, histopathological changes occurring in the brain can be divided into two processes: first, the formation of extracellular plaques (senile plaques) by deposition of β-amyloid peptides (Aβ), which is formed by amyloidogenic cleavage of amyloid precursor protein (APP) by β and γ secretases. Secondly, the formation of neurofibrillary tangles (NFT) that originate from paired helical filaments of phosphorylated protein tau (p-Tau) associated with microtubules [131]. Evidence shows that exosomes contain full-length APP and several proteolytically cleaved non-APP products, including Aβ; thus, they can act as vehicles for neuron-to-neuron transfer of such toxic proteins [132].

With regards to p-Tau, it should be noted that most of the tau proteins released into extracellular fluids are truncated tau, lacking ends that cause aggregation of this protein [133]. Therefore, full-length aggregation-capable tau is thought to be secreted into exosomes or axonal transmission. These processes may be the main vector of abnormal transmission of p-Tau [134], and occur in the midst of the dysfunction of the proteasomal and lysosomal system, common in AD, which leads the MVB to fuse with the plasma membrane instead of fusing with a lysosome for the degradation of its cargo. As a result, this leads to a greater release of exosomes in AD brains; these exosomes, as mentioned previously, contain APP, Aβ and p-Tau, possibly leading to a greater spread of the disease [130].

In parallel, interest in the study of exosomal cRNA has surged as more evidence of its regulatory function in target genes that could be involved in the pathogenesis of AD has been divulged [135,136,137,138,139]. For instance, miARNs 106a, 502c, -106b and 17-5p could potentially downregulate the expression of the *APP* gene, and decreased expression of miR-29a and miR-29b-1 in AD patients has been correlated to higher expression of the *BACE1* gene, which encodes an enzyme that limits the production of beta-amyloid peptide [136]. Other important ncRNAs, acting as PSEN1, PSEN2 and Tau regulators, have also been identified in patients with this devastating disease [137,138]. Long-lived individuals have been reported to have low mRNA expression levels of the *SIRT1* gene and high expression levels of miR-34a in peripheral blood [140]. Interestingly, increased expression levels this miRNA was reported in the temporal cortex of patients with AD [141]. Similarly, miR-34c expression is thought to increase in response to pathological stress [142]. Evidence suggests that stress-induced gene dysregulation promotes cognitive impairment [143]. Other miRNAs involved in the pathogenesis of AD are miR-146, which promotes pathogenic stimulation of innate immune and neuroinflammatory pathways, and miR-125b, which correlates with increased phosphorylation of tau [144].

Parkinson’s disease (PD), the second most common neurodegenerative disease after AD, has been associated with the loss of A9-type dopaminergic neurons that project from the substantia nigra in the midbrain to the dorsal striatum [145]. This process is characterized by the intraneuronal aggregation of the misfolded alpha-synuclein (α-syn) protein and the presence of Lewy bodies. α-syn is necessary for the release of neurotransmitters, since it facilitates the association of synaptic vesicles with the SNARE complex [146]. Under normal conditions, α-syn is degraded by the lysosomal autophagy system and the proteasomal pathway. This is evidenced in subjects carrying mutations in the *PARK-LRRK2* gene, showing a defective lysosomal machinery. Consequently, the α-syn contained in MVBs is diverted towards the plasma membrane [147]. In this way, the exosomal machinery serves as an auxiliary mechanism to spread the early molecular changes of PD to other cells.

Some ncRNAs have been implicated in the PD pathogenic network [148]. For example, increased expression of miR-126 has been shown to lead to neurotoxicity [149], and overexpression of miR-16-1 negatively regulates α-synuclein aggregation [150]. Likewise, decreased expression of miR-133b and miR-153 have also been associated with the pathogenesis of PD; the former regulates RhoA, an axonal growth inhibitor, with the consequent induction of axonal growth, and also inhibits the expression of α-synuclein [151]. While miR-153 reduces the activation of p38 and prevents apoptosis induced by neuroinflammation [152].

### 5.3. Exosomes in Cardiovascular Diseases

The role of exosomes in cardiovascular and metabolic diseases shares characteristics with their role in cancer. Emerging evidence suggests that exosomes mediate a range of paracrine signals within the cardiovascular system [153], such as between the vascular endothelium and the smooth muscle [154], between heart fibroblasts and cardiomyocytes [155] and between the vascular smooth muscle cells [156].

Exosomes of cardiovascular origin are also present in the pericardial fluid [157] and in blood [158,159], suggesting a probable involvement in endocrine signaling. In this regard, in cardiovascular conditions, such as cardiac fibrosis, atherosclerosis, heart failure, myocardial infarction and cardiac hypertrophy [160], circulating vesicles can change not only in number, but also in content.

In cardiac fibrosis, which occurs after injury, especially myocardial infarction, the molecular mechanisms are mainly related to the transforming growth factor β (TGFβ) pathways, the IL-11 signaling pathway and the nuclear factor-κβ and the Wnt pathways [161]. Therefore, any bioactive substance transported in exosomes that could interact with these pathways will participate in the pathogenesis of this heart condition. This is the case of WNT3a, WNT5a and tumor necrosis factor α (TNF-α) transported in exosomes. WNT3a inhibits the activity of glycogen synthase kinase 3β (GSK3β), induces nuclear translocation of β-catenin, and activation of activated T-cell factor (TCF)/lymphoid-enhancing factor (LEF) in cardiac fibroblasts, while WNT5a promotes the non-canonical Wnt and ERK1/2 pathways, as well as the c-Jun N-terminal kinase pathway, inducing the production of IL6 [162].

In atherosclerosis, injury to the arterial endothelium is recognized as an initial event of the disease. Endothelial cells have been shown to release and take up vesicles, especially under oxidative stress. Therefore, exosomes could participate in the intercellular communication process regulating the integrity of the intima. It has been reported that proatherogenic vascular miRNAs (i.e., miR-712 and miR-205) down-regulate tissue metalloproteinase inhibitor (TIMP3), activate matrix metalloproteinases (MMP) and lead to inflammation, vascular hyperpermeability and smooth muscle cell migration to areas of vascular hyperpermeability [163]. Other proatherogenic exosomal miRNAs are miRNA-23b, which suppresses endothelial cell proliferation; miRNA-92a and miRNA-205, which increase inflammation, miRNA-155, which decreases the synthesis of nitric oxide and miRNA-221 and miRNA-222, which promote the calcification of vascular smooth muscle cells [160].

Turbulent coronary blood flow and the resulting oxidative stress also negatively regulate anti-inflammatory and anti-atherogenic miRNAs, such as miRNA-10a, miRNA-19a, miRNA-23b, miRNA-101, miRNA-143 and miRNA-145, inhibiting cell activation as well as endothelial-NF-κB and vascular endothelial repair [163]. Cardiomyocyte hypertrophy, on the other hand, is a common response to increased hemodynamic load of the heart (such as arterial hypertension or valve stenosis), myocardial injury or neurohormonal stress. Hypertrophy allows the myocyte to generate more work, with increased cardiac pump function. This compensatory action, however, is at some point overwhelmed by biomechanical stress, which increases heart failure, leading to high morbidity and mortality [164].

Reports suggest that angiotensin II stimulates cardiac fibroblasts to release exosomes; these are subsequently taken by cardiomyocytes, where they can enhance cardiac hypertrophy by altering gene expression [165]. Yu et al. observed a greater release of exosomes in hypertrophic cardiomyocytes, and that these exosomes triggered the secretion of IL-6 and IL-8 as measured by the regulation of the Argonaut 2 (*AUG2*) protein gene [166]. Likewise, miR-21-3p, whose target genes encode the Sorbin And SH3 Domain Containing 2 (SORBS2) and PDZ And LIM Domain 5 (PDLIM5) proteins through a mechanism that is not yet fully understood, is capable of inducing cardiomyocyte hypertrophy [155].

### 5.4. Exosomes in Infectious Diseases

Exosomes can carry pathogenic molecules that play an important role in infectious processes. These molecules can be disseminated through vesicles derived by infected cells or by the microbial cells themselves [167,168]. Their participation in the pathogenesis of these diseases may contribute to the spread of infection or to the induction of inflammation by activating receptor cells with their contents [54].

The role of these nanovesicles has been extensively studied in viral infections, reporting common properties between exosomes and viruses. For example, it is known that the ESCRT machinery is involved in the generation of both these vesicles and viruses and that the exosomal content is modified after infection [169]. Thus, exosomes can facilitate viral replication and transmission by functioning as carriers of viral genetic elements, viral proteins or regulatory elements [169].

In the specific case of HIV, it has been reported that tetraspanins CD63 and CD81, enriched in exosomes, participate in viral budding and dissemination [167]. It has also been shown that exosomes carry HIV-1 proteins involved in the viral replication cycle, such as GAG and negative regulatory factor (Nef). The latter induces T cell apoptosis in vitro, a key feature of HIV infection [170]. Other examples include the transport of viral genome and replication proteins through exosomes released by cells infected by Rabies, Zika and hepatitis C viruses [67,171,172], facilitating their spread. 

In this regard, it has been hypothesized that exosomes play an important role in the propagation of the SARS-CoV-2 virus as well as in the induction of inflammation that contributes to organ dysfunction in some patients suffering from COVID-19 [173]. On the one hand, it is known that the spike protein shares a high degree of structural homology with angiotensin converting enzyme 2 (ACE2), which is transferred to recipient cells via exosomes [174]. It is presumed that as ACE2 is packaged into exosomes, components of SARS CoV-2 virus, such as its viral genome and proteins, may be packaged into exosomes. Barberis et al. [175] revealed the presence of SARS-CoV-2 RNA in the circulating exosome load of critically ill and non-critically ill patients. The authors found between 15 and 88 copies of genetic material/10 µL with no significant differences between the two groups, and it was not detected in healthy subjects. On the other hand, Kwon et al. demonstrated that pulmonary epithelial cells susceptible to SARS-CoV-2 infection can secrete exosomes containing viral components that are then taken up by cardiomyocytes, resulting in a critical indirect route of transmission and potentially leading to cardiac dysfunction through an increase in inflammatory markers [176]. This allows us to speculate on the possible role of exosomes in the pathogenesis of SARS-CoV-2 especially in virus-induced sepsis [177].

Alterations in number, content and function of exosomes have been demonstrated during sepsis in general [178]. These nanovesicles carry increased levels of cytokines, pathogen-associated molecular patterns (PAMPs) and damage-associated molecular patterns (DAMPs) [179]. PAMPs are molecules derived from microorganisms, whereas DAMPs are molecules released from stressed or dying cells. PAMPs and DAMPs induce inflammation by binding to pattern recognition receptors (PRRs) causing multiple organ dysfunction [180]. In COVID-19, for example, an exosome proteomic profile was reported in critically ill and non-critically ill patients. By using bioinformatics tools, it was possible to identify that these proteins are mainly related to immune response, inflammation and coagulation [175]. Indeed, tenascin-C (TNC) and fibrinogen-β (FGB), which stimulate proinflammatory cytokines through the nuclear factor-κB (NF-κB) pathway, are widely abundant in plasma exosomes of COVID-19 patients compared to healthy subjects [181].

## 6. Potential Role of Exosomes in Diagnosis of Diseases

Proteins and RNA transported by exosomes, which are released into circulation from healthy subjects and patients suffering from different diseases, can be measured and used as potential diagnostic markers, as summarized in Table 2 [182].

### 6.1. Exosomes in Cancer Diagnosis

Some microRNAs, enriched in TDEs, have been proposed as possible tumor markers [183,184]. Skog et al., showed that circulating exosomes of patients with glioblastoma carried large amounts of EGFRvIII mRNA, which could be potentially be measured to support the diagnosis of glioblastoma [185]. This method could be considered a “fluid biopsy”, and avoids the need for a surgical procedure to remove brain tissue for the detection of EGFRvIII. Similar cases were tested with molecules, such as human epidermal growth factor receptor 2 and proteoglycan 1 for the diagnosis of lung cancer and benign pancreatic disease, respectively [186,187].

In breast cancer (BCA), miR-7641 was identified to promote tumor cell progression and metastasis through intercellular communication. In particular, miR-7641 levels were significantly elevated in the plasma of patients with BCA compared with patients without metastasis. Thus, miR-7641 could be considered as a promising non-invasive diagnostic biomarker [188]. In addition, the combination of four miRNAs (miR-1246 + miR-206 + miR-24 + miR-373) have been shown to have a sensitivity of 98%, specificity of 96% and accuracy of 97% for BCA detection [189]. Furthermore, a ultrasensitive method was recently proposed to analyze the quantitative profile of surface protein biomarkers of exosomes by integrating mass spectrometry, imaging and gold nanoparticle-based signal amplification [190]. The authors reported that multiple biomarkers can be quantitatively detected, with a detection limit of up to 50 exosome particles. As a proof-of-concept, exosomes secreted by different BCA cell subtypes (MCF-7 and MDA-MB231) were used. This system efficiently identified characteristic CD9, CD44 and epithelial cell adhesion molecule (EpCAM) surface proteins, demonstrating that it could be used for cancer diagnosis and cell phenotype characterization [190].

Studies have also proposed biomarkers for cervical cancer (CeCA). Indeed, the expression levels of exosomal miR-125a-5p in CeCA patients were significantly lower than in healthy controls (*p* < 0.001). The results of receiver operating characteristic (ROC) curve analysis showed that plasma levels of exosomal miR-125a-5p was a potential marker for CeCA (area under the curve [AUC] = 0.713). Furthermore, the diagnostic sensitivity and specificity of this miRNA were 59.1% and 84.2%, respectively [191]. On the other hand, increased expression of CircEIF4G2 was found in tissue from cervical lesions and in circulating exosomes, suggesting that these exosomes are secreted by CeCA cells. Therefore, CircEIF4G2 can be used as a marker for the diagnosis of cervical lesions [192].

Urinary exosomal biomarkers have also been proposed as a diagnostic alternative in PCa. For example, miR-532-5p in urine exosomes could be a potential biomarker for predicting biochemical recurrence, which is a poor prognostic predictor in patients with intermediate-risk PCa [193]. Significant down-regulation of miR-375 and up-regulation of miR-451a, miR-486-3p and miR-486-5p expression levels have been reported in urinary exosomes of PCa patients compared to healthy subjects. These urinary exosomals miRNAs differentiate between localized and metastatic PCa (AUC = 0.806), and patients with PCa from patients with benign prostatic hyperplasia, by using a panel combining miR-375 and miR-451a (AUC = 0.726) [194]. Similarly, miR-423-3p was shown to be associated with castration-resistant prostate cancer (CRPC), and the combination of miR-423-3p with prostate-specific antigen improved its prediction (AUC = 0.908), demonstrating that miR-423-3p can serve as a biomarker for the detection and prediction of CRPC [195].

For lung cancer, a diagnostic model based on the expression levels of two miRNAs (miR-1268b and miR-6075) has been recently proposed; this model yields sensitivity and specificity values of 99% regardless of histological type and stage of cancer, and its diagnostic rate was reported to markedly decreased after lung cancer resection [196]. Another study proposed exosomal miR-1246 and miR-96 as biomarkers of non-small cell lung cancer (NSCLC), as they could be shown to be significantly overexpressed in individuals with the disease. Furthermore, exosomal miR-96 was reported to be significantly correlated with radioresistant NSCLC and vascular invasion [197]. Likewise, Xian et al., observed that linc01125 loaded in circulating exosomes could identify subjects with NSCLC and was associated with poor overall survival [198].

### 6.2. Exosomes in Neurodegenerative Diseases Diagnosis

Some exosomal biomarkers have also been proposed for the diagnosis neurodegenerative diseases. Specifically, APP has been reported to be produced in early endosomes of neurons with subsequent excision of the amyloid-peptide β, this is loaded into the MVBs [215,216,217]. Thus, Rani et al. proposed a novel method based on nanoparticle tracking analysis (NTA) to directly correlate salivary exosome concentration with the progression from mild cognitive impairment (MCI) to AD. Furthermore, significant differences were observed when comparing salivary exosome concentration between healthy controls and the MCI and AD patient groups (*p* = 0.0023). The performance of their method was corroborated with the expression level of oligomeric Aβ and phosphorylated tau protein in salivary exosomes. In this sense, the concentration of oligomeric Aβ (*p* = 0.0198) and phospho-tau (*p* = 0.0325) were significantly higher in subjects with AD vs. subjects with MCI and healthy subjects [199].

On the other hand, levels of auto-lysosomal proteins, present in exosomes differentiate patients with AD from controls, and seem to predict AD pathology up to 10 years prior to clinical onset [218]. Likewise, exosomal ncRNAs BACE1-AS, 51A, 17A, NDM29, BC200, miR-135a, miR-34b, miR-125b and miR-130b [136,200,201,202,219,220,221,222,223,224] and the exosomal proteins GAP43, neurogranin, SNAP25 and synaptotagmin 1 and hemoglobin of neuronal origin have been proposed as early biomarkers of AD and MCI [203,204].

In PD, exosomal biomarkers have also been proposed for the diagnosis of disease. In particular, Jiang et al. proposed α-synuclein (α-Syn) and clusterin loaded in serum exosomes as potential biomarkers that might allow the differentiation of PD patients from other phenotypically similar neurodegenerative movement disorders [205]. Another study also reported that oligomeric α-Syn increased while STX-1A and VAMP-2 were significantly reduced in PD patients compared to healthy subjects (*p* < 0.001) [206]. Regarding the use of serum exosomal miRNAs, Barbagallo et al. observed that let-7d, miR-15b, miR-24, miR-142-3p, miR-181c, and miR-222 could be considered as potential biomarkers of PD [202]. Furthermore, salivary exosome-loaded miRNAs have also been proposed for PD diagnosis. In this regard, it was shown that miR-153 (AUC = 0.79, 95% confidence interval [CI] = 0.61–0.96) and miR-223 (AUC= 0.77, 95%CI = 0.59–0.95) were significantly decreased in the saliva of PD patients compared with healthy subjects [207].

Overall, the importance of these findings lies in the ability of exosomes of neuronal origin to cross the BBB [225], as they could become blood biomarkers that could provide information on the status of the central nervous system, which is highly isolated and therefore, not easily accessible.

### 6.3. Exosomes in the Early Detection of Pregnancy Complications 

Analysis of exosomal content has also been proposed for early prediction or diagnosis of some pregnancy complications and fetal development disorders [226]. For example, in preeclampsia, exosomes secreted by trophoblasts may contain miRNA, DNA and proteins that could help predict its onset much earlier compared to blood proteins, including soluble FMS-like tyrosine kinase 1, endoglin and placental growth factor, which only do so just before the onset of the disease [227]. In this regard, some researchers have shown that neprilysin, a protein contained in placental-released extracellular vesicles [228], and the miRNAs hsa-miR-486-1 and hsa-miR-486-2-5p are elevated in women with preeclampsia compared to a group of healthy pregnant women [229]. In contrast, placental protein 13 is decreased [230].

Li et al. [208] analyzed the differential expression of exosomal miRNA in plasma of 20 patients with preeclampsia, 20 women with fetal growth restriction (FGR) and 20 with a healthy pregnancy. They found that seven miRNAs were differentially expressed in exosomes of women with preeclampsia and controls. Among these, miRNA-153, miRNA-325, miRNA-342-3p and miRNA-653-5p were overexpressed, and miRNA-222-3p, miRNA-224-5p and miRNA-532-5p were found decreased in circulating exosomes of pregnant women with preeclampsia. In contrast, no significant difference in miRNA expression levels was observed between women with FGR and controls [208].

Another study showed lower expression of miR-23a-3p, miR-125b-2-3p, miR-144-3p, miR-192-5p, miR-205-5p, miR-208a-3p, miR- 335-5p, miR-451a, miR-518a-3p and miR-542-3p and higher expression of let-7a-5p, miR-17-5p, miR-26a-5p, miR-30c-5p, miR -141-3p, miR-199a-3p, miR-221-3p, miR-584-5p, miR-744-5p and miR-6724-5p in exosomes isolated from patients with preeclampsia compared to healthy women [231]. These miRNAs are important in signaling pathways related to disease pathogenesis. For example, miR-525e5p is able to suppress vasoactive intestinal peptide, a potent anti-inflammatory factor. In addition, miR-526b regulates the expression of matrix metalloproteinase-1 and hypoxia-inducible factor 1-alpha. Finally, miR-1269 controls the expression of the *FOXO1* gene as a critical factor in endometrial stromal decidualization and the implantation process [232].

In the case of gestational diabetes mellitus (GDM), circulating exosomes have been identified to increase in number in a pregnant woman with this metabolic disorder compared with a healthy pregnant woman. Additionally, exosomal load, especially miRNAs, are also often altered and therefore can serve as potential biomarkers [226]. Zhang et al., concluded that miRNA-125b and miRNA-144 have excellent diagnostic value for GDM, reporting that miRNA-125b was down-regulated (*p* <0.001), whereas miRNA-144 was up-regulated (*p* <0.001) in circulating exosomes and placental tissue of women with GDM. Levels of miRNA-144 in circulating exosomes were negatively correlated with body mass index before pregnancy (*p* = 0.018) and before delivery (*p* = 0.039), and positively correlated with blood glucose at 1 h, estimated using an oral glucose tolerance test (*p* = 0.044) [209].

On the other hand, Herrera-Van-Oostdam et al. isolated and purified placental exosomes from urine samples during the first, second and third trimester of gestation, and measured the expression profile of miR-516-5p, miR-517-3p, miR-518-5p, miR-222-3p and miR-16-5p by RT-qPCR. In this study, all miRNAs were found to be negatively regulated in patients with GDM in the third trimester of gestation and to affect several metabolic pathways closely associated with the pathophysiology of GDM [210].

### 6.4. Exosomes in Cardiovascular Diseases Diagnosis

Acute myocardial infarction (AMI) is one of the most frequent causes of death worldwide. Although considerable progress has been made in its diagnosis, there is still an urgent need for new biomarkers for its prevention and treatment. Recently, Guo et al., identified specific plasma exosomal miRNAs with biomarker potential for early detection of AMI [211]. Specifically, hsa-let-7i-5p, hsa-miR-143-3p, hsa-miR-1180-3p and hsa-miR-3615 miRNAs were found in large amounts in circulating exosomes. Furthermore, these miRNAs and hsa-miR-106b-5p, hsa-miR-17-5p and hsa-miR-1273h-3p correlated linearly with progression from coronary artery disease to AMI. In addition, Zheng et al., demonstrated that circulating exosomal lncRNAs ENST00000556899.1 and ENST00000575985.1 are elevated in patients with AMI, and may function as potential biomarkers to predict the prognosis of AMI patients. Interestingly, ENST00000575985.1 showed significant correlation with clinical parameters, including inflammatory biomarkers, prognosis indicators and markers of myocardial damage. This lncRNA also showed a positive association with the risk of future heart failure in patients with AMI [213].

Another study explored the role of miRNAs miR-126 and miR-21, and phosphatase and tensin homologue (PTEN) contained in blood exosomes as potential biomarkers of AMI and unstable angina pectoris (UA) [212]. Levels of the circulating exosomal miR-126 were positively correlated with the severity of coronary artery stenosis in patients with UA (*r* = 0.7137, *p* <0.0001) and AMI (*r* = 0.6028, *p* = 0.0003), assessed by the Gensini score [212].

### 6.5. Exosomes in COVID-19 Diagnosis

Some studies have explored the analysis of exosomal content for the diagnosis of COVID-19 and for the classification of patients according to disease severity. These analyses include proteomic profiling, quantification of exosomes according to phenotype, and RNA analysis [175,214,233].

It has been reported, for example, that fibrinogen alpha, beta and gamma chains contained in exosomes are able to discriminate between patients with COVID-19 and healthy subjects with an AUC value of 0.94 (sensitivity: 86%; specificity: 97%), 0.90 (sensitivity: 92%; specificity: 86%) and 0.93 (sensitivity: 83%; specificity: 91%), respectively. In addition, fibronectin, complement subcomponent C1r and serum amyloid P component showed an AUC value of 0.92 (sensitivity: 94%; specificity: 82%), 0.93 (sensitivity: 89%; specificity: 82%) and 0.91 (sensitivity: 89%; specificity: 82%), respectively [175]. Likewise, 114 exosomal cRNAs and 10 exosomal ncRNAs differentially expressed in subjects with COVID-19 and healthy subjects have been identified. The gene corresponding to these cRNAs was found to be mainly involved in the regulation of immunity and inflammation, cell cycle and apoptosis [233]. This differential expression could provide a new way to explore the pathogenesis of COVID-19 and be taken into account when postulating diagnostic markers.

Regarding the classification of patients according to disease severity, Kudryavtsev et al. propose a quantitative analysis of various phenotypes of plasma extracellular vesicles based on differential centrifugation, immunostaining and highly sensitive multicolor flow cytometry. The authors found that plasma levels of CD235a+ and CD14+ exosomes were significantly increased in patients with moderate infection, whereas CD8+ and CD19+ exosomes were decreased compared with the group of healthy subjects. On the other hand, patients with severe infection had lower CD4+, CD19+ and CD146+ levels compared with healthy individuals [214].

Likewise, it has been reported that critically ill patients carry higher levels of CRP, alpha-1-acid glycoprotein 1 and 2, chemokine ligand 7, and zinc-alpha-2-glycoprotein in their exosomes, and lower levels of coiled-coil domain-containing protein 34, and complement component 4 binding protein alpha, compared with non-critically patients [175].

## 7. Potential Role of Exosomes in the Treatment of Diseases

The discovery of exosomes as natural carriers of proteins and RNA has propelled a great interest in the field of pharmacology and therapeutics. Exosome translational research has showed that these vesicles could be used as diagnostics biomarkers of disease, but also could be taken advantage of for the therapeutic administration of medicinal RNAs, peptides and synthetic drugs [234] Recent studies, showing the potential role of exosomes in the treatment of diseases, are summarized in Table 3. However, there are still technical challenges to overcome, and we are still far from being commonly used in clinical practice. Liposomes or synthetic vesicles have been used as an artificial drug delivery vehicle for over 40 years [235]. Exosomes offer the potential for a natural drug delivery system and have several advantages over liposomes [235,236], such as their ability to evade the immune system and their phagocytosis by macrophages, due to their expression of molecules, such as CD55 and CD59 [237]. Furthermore, exosomes express a complex matrix of proteins within their membrane that can be modified to include ligands on their surface, allowing them to be directed to specific target cells [236,237]. Moreover, these vesicles can to some extent, cross over the blood–brain barrier (BBB), which is not the case for some drugs [238]. Distribution to the CNS could be achieved for instance, by the incorporation of a peptide of a rabies virus glycoprotein into the exosome membrane. This has also been shown when LAMP2b, a protein ligand fused to a lysosome-associated membrane, successfully targets the exosomes into the brain of mice [239,240]. Similarly, research studies in cancer have shown that the administration of drugs through the BBB leads to a decrease in brain tumor markers [241].

### 7.1. Potential Role of Exosomes in Cancer Treatment

Numerous studies have aimed to evaluate exosomes as potential delivery systems for chemotherapeutics with anticancer properties, and as biological reprogrammers of cancer cells [242]. Thus, Han D, et al. compared the antitumoral efficacy of paclitaxel encapsulated by electroporation in natural killer cell-derived exosomes (PTX-NK-Exos) as compared with free paclitaxel (PTX). They found that the PTX-NK-Exos drug-loading system at the same dose had an inhibition rate in MCF-7 cells compared with free PTX. PTX-NK-Exos exerted an antitumor effect by inducing positive upregulation of Bax and caspase-3 in the apoptotic signaling pathway in these tumor cells [243]. Similarly, it has been shown that cisplatin loaded into M1 mononuclear macrophage exosomes by sonication increased its cytotoxicity 3.3-fold in human ovarian cisplatin-resistant cancer cell lines (A2780/DDP) and 1.4-fold in cisplatin-sensitive A2780 cells compared to traditional chemotherapy. Analysis concluded that cisplatin-loaded M1 mononuclear macrophage exosomes are a potentially powerful new tool for the delivery of chemotherapeutics, even in drug-resistant cells [244].

Treatment for tumors in the CNS represents a major challenge, among other reasons, due to the impermeability of the blood-brain barrier (BBB) that hinders drug access to the tumor. Such is the case of malignant glioma, which is usually resistant to chemotherapy [245]. In vitro and in vivo experiments have shown the ability of exosomes secreted by neutrophils (NEs-Exos) to cross the BBB. NEs-Exos have been loaded with doxorubicin (DOX) and in in vivo zebrafish and mouse models of glioma, it has been showed that NE-Exos carrying the drug responded chemotactically to the tumor inflammatory stimuli, rapidly penetrated the BBB, migrated to the brain and targeted infiltrating tumor cells, effectively restraining tumor growth. Based on these results, the authors confirmed that NEs-Exos have a chemotactic function similar to that of neutrophils and are able to cross the BBB, representing a new DOX delivery platform for the treatment of glioma and other solid tumors; however, this also opens the door for NEs-Exos to be used for the treatment of other brain diseases [246].

In recent years, exosomes have also been used experimentally as cancer immunotherapy treatments. They have been proposed as cancer vaccines due to their ability to carry antigens and MHC-peptide complexes, and to promote helper T-cell immune responses [273]. Programmed cell death ligand 1 (PDL1) is known to be expressed on the surface of tumor cells, immune cells and other cells within the tumor microenvironment, so PDL1 interacts with the programmed cell death protein 1 (PD-1) of T cells inhibiting their activation and antitumor immune response [274]. Tumor cells achieve immune escape and tumor growth is favored. Thus, anti-PD-L1 (aPD-L1) antibodies have been used to prevent PD-1 binding and partially restore T-cell activity [275].

However, therapy with these antibodies may be hampered by the polarization of macrophages within the tumor microenvironment (TME) into M2 tumor-associated macrophages (TAMs), which suppress antitumor immune responses and promote tumor growth by releasing anti-inflammatory cytokines and angiogenic factors [276]. Thus, Choo YW, et al., investigated whether repolarization of these macrophages can enhance the anticancer efficacy of aPD-L1. To this end, they used M1 macrophage-derived exosome mimetic nanovesicles (M1NV) to repolarize M2 TAMs into M1 macrophages, resulting in the release proinflammatory cytokines and the induction of antitumor immune responses. Results showed successful polarization of M2 macrophages to M1 macrophages in vitro and in vivo. Importantly, the combined injection of M1NV and aPD-L1 further reduced tumor size compared to injections of M1NV or aPD-L1 separately [253].

On the other hand, T cells expressing a chimeric antigen receptor (CAR) have been genetically engineered and have become a powerful and innovative therapeutic option for cancer patients. However, they have the caveat of dose-dependent systemic toxicity. It was shown that exosomes derived from CAR-expressing T cells (CAR-T) targeting mesothelin (MSLN) maintained most of the characteristics of the parental T cells, including surface expression of CARs and CD3. These CAR-bearing exosomes were also observed to significantly inhibit the growth of endogenous and exogenous MSLN-positive triple-negative breast cancer (TNBC) cells. Most importantly, inhibition occurred without the in vivo toxicity effects expected with the use of CAR-Ts. Thus, the use of CAR-T cell exosomes has great therapeutic potential against MSLN-expressing TNBC [254].

Finally, it has been identified that exosomes can control tumor growth due to certain specific proteins and RNAs that are transferred to malignant cells. This shows the role of exosomes as biological reprogrammers of tumor cells [242]. It has been shown that the exosomal transfer of miR-139-5p into bladder cancer cells has role in regulating tumorigenesis. miR-139-5p is down-regulated in bladder cancer, and acts by reducing the expression of the polycomb repressor complex 1 (PRC1) gene. It was demonstrated that upon exosomal miR-139-5p transfer into bladder cancer cells, cell proliferation, migration and invasion potentials were inhibited [255]. The same effect was evidenced by transfecting miR-381 loaded on exosomes isolated from adipose tissue-derived mesenchymal stem cells into TNBC cells. These significantly reduced the expression of genes and proteins related to epithelial-to-mesenchymal transition (EMT) and promoted apoptosis in vitro [256].

Other exosomal miRNAs that inhibit cancer cell proliferation, migration and invasion are miR-140-3p which targets BCL9 and BCL2; and miR-5100, which regulates the expression of CXCL12, thus inhibiting the CXCL12/CXCR4 axis, which is used by some cancer cells to spread to regional nodes of the primary tumor [257,258].

Additionally, a recent review cites studies that identified miR-1249, miR-126, miR-27b, 520a, miR-590-5p and miR-622 as inhibitors of angiogenesis in colorectal cancer (CRC). These miRNAs inhibit vascular endothelial growth factor A, whose elevated levels are closely related to CRC invasion and metastasis, through different pathways [259].

### 7.2. Potential Role of Exosomes in the Treatment of Neurodegenerative Diseases

As already mentioned, the therapeutic potential of exosomes in neurodegenerative diseases is enhanced by the fact that exosomes are able to cross the BBB. Therefore, exosomes may be a suitable tool for neurodegenerative disease therapy due to their ability to deliver nucleic acids and proteins to de CNS with specificity [277]. For example, it is known that enzymes that degrade Aβ peptide can be found in exosomes, among them are neprilysin and insulin degrading enzyme (IDE). Accordingly, their internalization leads to the reduction of extracellular and intracellular Aβ levels [278]. For this reason, efforts have been directed to the design of strategies to target exosomes loaded with these proteins to the brain. Kuwajima et al. transfected a plasmid vector encoding green fluorescent protein-tagged neprilysin into mouse C2C12 myotube cells. They then extracted exosomes released from these cells and labeled them with an infrared dye. These tagged-exosomes were injected into the mouse masseter muscle to track their movement to the hippocampus via the trigeminal nerve. The results indicated exosomal transport of neprilysin into the hippocampus where the protein, but not its mRNA could be detected [247].

Another molecule that has been proposed for neuroprotection is quercetin (Que), which can prevent the formation of insoluble neurofibrillary tangles (NFT) and improve cognitive and functional symptoms of Alzheimer’s disease (AD). Delivery through plasma exosomes (Exo) loaded with Que (Exo-Que) was found to improve the brain targeting and bioavailability of Que. Furthermore, compared to free Que delivery, exo-Que improved AD symptoms by inhibiting cyclin-dependent kinase 5-mediated phosphorylation of Tau and reducing the formation of NFTs, suggesting it may have therapeutic potential in AD [248].

Several studies have shown the beneficial effect of exosomal miRNAs in neurological disorders. For instance, analysis of exosomal miRNAs derived from mesenchymal stem cells have shown to improve different brain disorders pathologies, including AD (miR-21, miR-29b and miR-146a), subarachnoid hemorrhage (miR-21 and miR-193b) and traumatic brain injury (miR-216a) [249]. In the case of Parkinson’s disease (PD), it was shown that treatment with miR-188-3p-enriched exosomes suppressed autophagy and pyroptosis. This miRNA targets NACHT domain-containing protein 3 and protein kinase 5 of cell division [250]. Other RNAs that may have therapeutic potential for the treatment of PD are miR-7, whose expression enables normal development, physiology and neurogenesis in the central nervous system, and also maintains alpha-synuclein (α-Syn) at the physiological level [251] and miR-30a-5p, which contributes to the pathogenesis of PD by regulating ubiquitin-mediated degradation of glutamate transporter 1. Deletion of miR-30a-5p has been shown to ameliorate motor deficits and pathological changes, such as astrogliosis and reactive microgliosis in a murine model [252].

### 7.3. Potential Role of Exosomes in the Treatment of Cardiovascular Diseases

There is a potential role of exosomes in cellular conditioning after acute myocardial infarction (MI). Some studies have shown that miRNA-21-5p loaded in cardiac telocyte exosomes were able to silence the cell death-inducing p53 target 1 gene (Cdip1), which normally induced cell death. Consequently, activated caspase-3 appeared to be downregulated, inhibiting apoptosis of recipient endothelial cells under ischemic and hypoxic conditions, which facilitated angiogenesis and regeneration after MI [260]. Likewise, exosomes secreted by cardiac-derived progenitor cells (CDC) transferred to macrophages after reperfusion were associated with a reduction of the infarct size in an animal model or cardia ischemia. These exosomes are enriched with several miRNAs (including miR-146a, miR-181b and miR-126), among them, miR-181b which reduces protein kinase C δ transcript levels in macrophages and facilitates their polarization promoting cardioprotective effects [261]. CDCs have also been modified to express βARKct (βARKct-CDC), an inhibitor of the G protein-coupled receptor kinase 2 (GRK2). When added to cardiomyocytes in culture, βARKct-CDC protected them against hypoxia-induced apoptosis. Furthermore, in an in vivo model, after MI injury or acute catecholamine toxicity, these exosomes also showed significant protection against ischemic injury, improving cardiac function [262].

Although exosomal content offers great potential in the diagnosis and treatment of different diseases, a better understanding of the biological functions, as well as verification of the sensitivity and specificity of each biomarker under well-defined conditions are needed before conclusions can be drawn. It is also necessary to overcome some challenges related to the use of exosomes in drug delivery, such as drug loading, their cellular absorption, drug release and in vivo distribution.

### 7.4. Potential Role of Exosomes in the Treatment of Infectious Diseases

In infectious diseases, exosomes and other extracellular vesicles not only mediate the transfer of virulence factors to promote the survival of the microorganism and the disease, but also carry pathogen-derived molecules or immunomodulators that favor the elimination of the microorganism and the balance of the immune response [167,168]. Thus, exosomes have been evaluated as potential carriers of molecules to control or prevent infection [168]. In this section we will review the role of these vesicles in the control of bacterial infections, sepsis and COVID-19.

MSCs have been shown to have the ability to fight infections through direct microbicidal properties and/or by modulating the immune response [263,279], as they constitutively express antimicrobial peptides (AMPs), such as cathelicidin LL-37, human β-defensin-2 (hBD-2), hepcidin and lipocalin-2 (Lcn2). These peptides bind nonspecifically to the cell wall of Gram-positive and Gram-negative bacteria and destroy them [263]. Compared to antibiotics, AMPs have the following advantages: lower tendency to generate resistance, a low propensity to develop toxicity, better control of infection by intracellular bacterial pathogens as opposed to the ineffectiveness of antibiotics, anti-biofilm effects and immunomodulatory properties, induce cytokine production, immune cell activation and differentiation [279].

Exosomes secreted by MSCs carry these AMPs and have the property to interact with infected cells to control infection, making them promising candidates and a future alternative to conventional antibiotics [279]. However, there are currently some limitations related to the low reproducibility and standardization of MSCs from different sources, as well as obtaining their exosomes and/or purification of secreted antimicrobial peptides for use in cell-free therapy.

Exosomes have also become an important target and focus for the sepsis treatment [280]. For example, miR-27b loaded on MSC-derived exosomes inhibit the development of sepsis by regulating Histone demethylase Jumonji D3 and inactivating the nuclear factor κB (NF-κB) signaling pathway, thereby decreasing the production of proinflammatory cytokines [267]. In addition, exosomal miR-21 plays an important role in renoprotection conferred by remote ischemic preconditioning (rIPC) of the limbs, proposed as a therapeutic strategy for sepsis-induced renal injury. This exosomal miRNA acts on the programmed cell death protein 4/NF-κB and PTEN/AKT pathways, exerting anti-inflammatory and anti-apoptotic effects [265].

Choi et al. [266] implemented a system to load a large amount of super-repressor IκB (srIκB) into purified exosomes (Exo-srIκBs), designed optogenetically (EXPLOR). srIκB is the dominant active form of IκBα and can inhibit NF-κB translocation in the nucleus. It was shown that that intraperitoneal injection of exo-srIκBs attenuates mortality and systemic inflammation in septic mice.

Regarding COVID-19, a subset of patients with severe disease is known to develop an excessive host immune response compatible with a “cytokine storm syndrome”, leading to acute respiratory distress syndrome (ARDS), acute lung injury, multiple organ failure and death [281]. It is for this reason that, as in sepsis, exosomes have been proposed as a therapeutic option for the immunomodulatory treatment of COVID-19 [282].

Some clinical trials involve the use of AeroLyzed or intravenously delivered exosomes https://www.clinicaltrials.gov/ct2/results?cond=covid-19&term=exosomes&cntry=&state=&city=&dist= (accessed on 9 August 2021). For example, some of them aim to evaluate the safety and efficacy of CD24-overexpressing exosomes to prevent clinical deterioration in patients with moderate to severe disease [267,268,269], as they can directly suppress cytokine storm. CD24 is a key player in the vast majority of human cancers and also plays an important role in the control of homeostatic T-cell proliferation. Thus, CD24 may negatively regulate inflammation. T cell-derived exosomes are also being studied for the treatment of pneumonia in patients with early stage COVID-19. A clinical study [270] evaluating the safety and efficacy of these exosomes (CSTC-Exo) administered by aerosol inhalation. Similarly, investigations for the treatment of severe COVID-19 pneumonia [283,284] have been developed based on the promising results of a pilot study [285] aimed at improving their safety and therapeutic outcomes following exosome inhalation.

Other clinical trials deliver exosomes intravenously. Thus, the efficacy of MSC-derived exosomes [286] as well as cardiosphere-derived cells (CDC) [287] is also being evaluated. CDCs are known to secrete numerous bioactive elements (growth factors, and exosomes) that potentiate the therapeutic benefits of cell therapy. Immunomodulatory, antifibrotic and regenerative effects are expected in both clinical trials.

Currently, the results of the use of MSC-derived exosomes (ExoFlo^®^) in a prospective non-randomized cohort study showed safety in the improvement of pneumonia, negatively regulating the cytokine storm and reconstituting the immunity of patients with COVID-19 [271]. Likewise, the use of Zofin (previously known as Organicell Flow), an acellular product developed from human amniotic fluid, was also tested in patients with moderate to severe COVID-19. This product contains more than 300 growth factors, cytokines and chemokines, as well as other extracellular vesicles (including exosomes) derived from amniotic and epithelial stem cells. After 28 days of intravenous administration, researchers reported no adverse events associated with the therapy, and that those patients showed improvement in their clinical status, including inflammatory biomarkers [272].

## 8. Methods for the Isolation, Characterization, and Analysis of Exosome Content

In order to expand the usage of exosome analysis in the clinical setting, understanding and optimization of techniques for their assessment becomes essential. A summary of the techniques used for isolation and characterization of these nanovesicles, as well as for the identification of exosomal content, and the methods used for drug delivery via exosomes (i.e., drug loading and release) is presented as follows.

### 8.1. Methods for Exosome Isolation

There are several methods for the exosome isolation either from cell media or biological fluids based on their shape, density, size, or surface proteins. Each method has advantages and limitations. Thus, selecting the proper technique may not be an easy task [288]. In what follows, we will discuss the most important aspects of each method.

#### 8.1.1. Ultracentrifugation

Ultracentrifugation is considered the gold standard for exosome isolation, and is based on the separation of the different components of the sample (cell media or bodily fluid) according to their density [288]. This is a two-step method. First, samples are centrifuged sequentially at various speeds (300, 2000 and 10,000× *g*) in order to remove cells, cell debris, apoptotic bodies and microvesicles, respectively [289]. The remaining supernatant contains a relatively high concentration of exosomes, although at this point it may be still contaminated with some microvesicles, lipoproteins and other protein aggregates [290]. Secondly, the precipitation of the exosomes is achieved by centrifuging the supernatant at 100,000× *g* for 70 min—2 h [289]. To increase its purity, the resulting pellet can be resuspended in phosphate buffered solution (PBS) and ultracentrifuged again. Seventy percent of the exosome fraction obtained by this method consists of vesicles of 50–150 nm in diameter [291].

Nevertheless, there are various factors that could interfere with the efficiency of the method. These include centrifugation time, centrifugal force, rotor type and sample viscosity, among others [292]. In this sense, it is recommended to adjust the centrifugation conditions according to the characteristics of the rotor used. It is also advisable to, if possible, reduce the viscosity of the sample, since the higher the viscosity of the suspension, the more difficult it will be for the exosomes to move through the sample and to precipitate [293]. Finally, possible disadvantages of this technique include the precipitation of lipoproteins of similar density to that of exosomes, the generation of exosomal aggregates [294], the duration of the procedure and the cost of the equipment used [295]. In this sense, changes to ultracentrifugation protocols have been implemented to further improve the purification process and the performance of this method, such as the use of iodoxynol or sucrose-density gradients [289]. A density gradient enables the exosomes to localize within the layer matching their specific density, while contaminants with different densities will migrate to other layers [295].

#### 8.1.2. Ultrafiltration

Filtration uses membrane filters that allow separation of exosomes from other sample components based on their molecular weights and sizes [296]. Larger particles are first removed by using 0.8 and 0.45 µm pore diameter filters. Consequently, the solvent and small molecules will be filtered through the membrane, while bigger particles will be trapped in the membrane. Smaller vesicles are then removed from the filtrate using membranes with smaller pores (0.22 and 0.1 µm) [293]. This protocol can be used as a complement to ultracentrifugation or gel filtration chromatography, although it can also be used as a stand-alone technique as well [296].

There are some advantages of ultrafiltration over ultracentrifugation. Ultrafiltration is less time consuming and avoids the need for expensive equipment. In addition, ultracentrifugation requires a centrifugal force of 100,000× *g*, which could damage the integrity of exosomes, whereas ultrafiltration requires a pressure of only 517.125 kPa [295]. This greatly reduces the probability of exosome rupture, which results in a higher yield per sample volume. Also, the final products obtained by ultrafiltration are not aggregated, which facilitates their use for further studies [295].

Among the limitations of ultrafiltration are the lack of purity of the final product, since it is difficult to exclude those particles or molecules with a similar diameter to the exosomes, and the non-specific binding of exosomes to the membranes, which could reduce the recovery rate [297].

#### 8.1.3. Precipitation with the Aid of Polymers: Coprecipitation

This method, also be referred to as coprecipitation, relies on the aggregation and further precipitation of exosomes at low centrifugation speeds using polymers. The most used polymer for this purpose is polyethylene glycol (PEG) [296]. To obtain concentrated preparations of exosomes that are free of proteins, lipoproteins and other impurities, pretreatment of the samples by centrifugation or ultrafiltration is required [296].

Polymer-aided precipitation is relatively easy and quick to perform compared with other methods, which makes it suitable when processing large amounts of samples. However, the purity and recovery rate are relatively low, and the polymer may be difficult to remove, interfering with the subsequent experimental analysis of exosomes [290]. A viable strategy to avoid these drawbacks is to combine this method with an immunoprecipitation assay or other exosome enrichment techniques to obtain pure exosome fractions [293].

#### 8.1.4. Immunoaffinity Chromatography

Immunoaffinity chromatography is a separation and purification technology based on the specific binding of antibodies to proteins expressed in the surfaces of exosomes [290]. These antibodies are coating magnetic beads [298] or ferric oxide nanocubes [299]. The antibodies used in this method usually recognize tetraspanins, heat shock proteins and MHC antigens [296], which are the most common markers to be expressed on almost all exosomes. However, additional markers can also be used to isolate vesicles derived from a specific cell type [289]. In this regard, when comparing immunoaffinity chromatography with previously described methods, it is evident that this method allows the isolation specific exosome subtypes with high purity, which is particularly important for further proteomics, lipidomics or RNAomics studies [300]. The main disadvantage associated with this method is that the exosomal yield is usually low since only antibody-recognized vesicles are captured. Also, it is possible that this method could skew further analyses towards specific subpopulation of exosomes, excluding the particles that do not express the particular marker recognized by the antibody. Thus, subsequent analyses have to be interpreted carefully Additionally, its high cost poses a barrier to its implementation in the clinical setting [296].

This technique can be combined with other methods based on size and density, such as microfluidic platforms, which are divided into six categories based on their capture mechanisms: passive structure-based affinity, immunomagnetic-based affinity, filtration, acoustic-fluidic, electrokinetic and optofluidic [301]. These methods have advantages, such as fast and efficient processing, requirement of smaller sample volumes and high level of purity [302]. However, it should be noted that the devices are complex and expensive [301,302]. Although techniques based on microfluidics are new technology with promising prospects, it is not yet considered a standardized method for the isolation of exosomes [302].

#### 8.1.5. Size Exclusion Chromatography (SEC)

This method is based on the separation of molecules that differ in their hydrodynamic radius, and uses a biofluid as the mobile phase and a porous gel filtration polymer as the stationary phase [303]. The radius is defined as the apparent size of a solvated/hydrated particle or vesicle [304], and the polymer can be cross-linked dextran, agarose, polyacrylamide or allyl dextran. The nature of the stationary phase allows for differential elution, where larger particles eluting first, then the smaller vesicles (exosomes) and finally the proteins that do not bind to the stationary phase [304]. This is because the larger the particle, the fewer pores it can pass through and therefore it will travel a shorter path to the end of the chromatography column, causing it to elute faster compared to the smaller particles. The shape of the vesicles is also considered when isolating them, since changes in shape may cause them to elute in a different phase than the intact exosomes. This results in higher purity, specificity and integrity, of the isolated exosomes [293]. It requires an average processing time of 20 min per sample, which is on the efficient side of exosome isolation techniques [305]. As a limitation, the yield may be low, but it can be compensated by using a large volume of mobile phase [306]. A low yield of mRNA and proteins obtained from vesicles isolated with this technique has also been described [307].

As mentioned in previous sections, apoptotic bodies can be considered part of the EV family. Nevertheless, since their origin differs from exosomes or microvesicles, it is common practice to exclude them from purified exosome preparations. For instance, most researchers would perform a series of low-speed centrifugations of the conditioned media or plasma/serum in order to “clear” it from larger vesicles or particles, before the isolation of exosomes by ultracentrifugation, ultrafiltration and/or SEC [289,305,308]. In some cases, filters with a pore size of 0.45 um can be used to retain apoptotic bodies, whose size could reach ~1 um, producing a filtrate that would be cleared of cell debris and apoptotic bodies [293].

Including apoptotic bodies in the exosome preparation can lead to altered or even false results. For instance, if a treatment increases apoptotic cell death, exosome preparations contaminated with apoptotic bodies could report increased concentrations of proteins, mRNAs or other components that are not part of exosomes, but rather remained in the apoptotic bodies after cell demise. In fact, as discussed in further sections of this review, most researchers test their samples for the presence of proteins usually found within exosomes, but also would look for proteins that are not commonly found in them, and whose presence may indicate contamination with cell debris or apoptotic bodies, such as Calnexin [309,310]. Apoptotic bodies have been largely seen as a contaminant of exosome preparations. However, growing evidence shows that apoptotic bodies may have a central role in the regulation of the immune response in the context of autoimmunity, cancer and sepsis [311,312].

### 8.2. Methods for the Characterization of Exosomes

After isolation, it is necessary to evaluate whether the isolated particles are in fact exosomes, as well as the level of purity. In this sense, exosome characterization methods are mainly divided into two types: external characterization (morphology and size detection) and exosomal marker proteins (membrane and intraluminal proteins).

#### 8.2.1. External Characterization. Detection of Exosome Morphology

Electron microscopy (EM) is necessary to characterize the morphology of exosomes since particles smaller than 300 nm are too small to be observed with optical methods [313,314]. When studying biological samples, two types of EM are widely used, namely transmission electron microscopy (TEM) and scanning electron microscopy (SEM) [315]. The former is considered the gold standard for studying exosome morphology.

With TEM, a two-dimensional image is obtained. Since the wavelength of the electron beam is shorter than the wavelength of visible light by three orders of magnitude, images are recorded with a resolution of 1 nm. In addition, immuno-gold labeling opens the possibility of detecting specific proteins within the exosomes [314]. Unfortunately, the benefits of high resolution can easily be outweighed by the disadvantages related to sample preparation, as they must be fixed and dehydrated prior to their observation by TEM. In addition, image acquisition is performed under vacuum conditions which can further alter the morphology of exosomes [315].

Under TEM, exosomes appear round, making it possible to determine their diameter. This could be used as a confirmation of exosome presence in the sample, if particles are within the reported sizes for exosomes. Furthermore, exosomes have a characteristic cup shape when observed by TEM. This shape is attributed to the collapse of the structure after dehydration [316].

Although SEM resolution is lower than TEM’s, SEM has emerged as an alternative for exosome characterization [316]. Instead of using a wide beam as in TEM, SEM uses a fine-tipped beam that scans the samples line by line. As a result, instead of a two-dimensional image, SEM scans the surface of the samples to provide a three-dimensional image of the exosomes [314]. Wu et al. reported that, unlike the cup shape of exosomes observed under TEM, SEM showed rounded, bulging exosomes without a central depression. Diameter of exosomes appears to be similar when observed with either TEM or SEM. [316]. Like TEM, samples analyzed under SEM are fixed and chemically dehydrated in several steps, which often introduces artifacts that change the apparent exosome morphology [317]. Moreover, in some cases, the electron beam can also cause damage to biological samples. To circumvent these issues, cryo-electron microscopy (cryo-EM), which includes a different protocol for sample preparation, could become an alternative [314].

Cryo-EM is a type of TEM, in which samples remain in their native aqueous environments during analysis. In this method, the sample is stored and studied on vitreous ice at the temperature of liquid nitrogen, so invasive steps, such as dehydration or fixation are omitted [289]. This avoids ultrastructural changes and redistribution of elements [314]. Melyanov et al. were able to visualize with cryo-EM a broad spectrum of extracellular vesicles of various sizes and morphology with lipid bilayers and vesicular internal structures [318].

#### 8.2.2. External Characterization: Exosome Detection According to Their Size

Nanoparticle tracking analysis (NTA) is a sophisticated method for measuring exosome concentration and size distribution [289]. Particles in suspension are injected into a measuring chamber where they are exposed to a laser beam. The movement of the particles over a certain period of time is then recorded by a highly sensitive camera mounted on an optical microscope. From the obtained video recording, the displacement of each particle is tracked and plotted as a function of time, which allows the calculation of the size distribution by applying the two-dimensional Stokes–Einstein equation to determine their hydrodynamic diameters [314]. The entire sample measurement process takes approximately 15 min and has better reproducibility than other methods for exosome characterization. NTA has high resolution and can detect vesicles with diameters from 30 to 1000 nm [289]. However, the lower limit can only be reached when measuring particles with a high scattering index. Exosomes range from 40 to 100 nm and may have a low scattering index [319], raising the concern that exosomes in the lower size range may not be observed as they might not scatted enough light to be detected [315].

Zhang & Lyden introduced a new method for both isolation and characterization of extracellular nanoparticles, including exosomes and exomers asymmetric-flow field-flow fractionation (AF4) [320]. With this technique, exosomes are separated based on their density and hydrodynamic properties. Exosomes flow through a forward laminar channel and, according to their Brownian motion, are classified into different populations. Smaller particles have higher diffusion rates and tend to move faster; conversely, larger particles have lower diffusion rates and tend to move slower [289,320]. This technique offers great advantages compared to other techniques, as it facilitates the successful separation of different subsets of exosomes and the identification of exomers. Moreover, the processing time is short (1 h) when compared to ultracentrifugation, and allows a number of subsequent characterizations that could provide information about the abundance of particles, particle size and purity [321,322]. However, loading capacity currently remains a drawback as large starting volumes are needed to produce sufficient material for downstream applications [321].

Tunable resistance pulse sensing (TRPS) is based on the movement of individual particles through a nanoscale pore. As the particle passes through the pore, the magnitude, duration, and frequency of the resistance pulses are detected and used to determine size, zeta potential and concentration [315]. It can detect vesicles with diameters from 50 to 1000 nm. Compared to NTA, TRPS has a higher size resolution and higher accuracy when measuring particle size distribution [289]. The main disadvantage of this technique is that TRPS measurements are susceptible to system stability problems, for instance, the pore can be blocked by particles, as well as to sensitivity problems, where particles are too small to be detected above the background noise [323].

One of the difficulties shared by the characterization methods according to the morphology and size distribution is that exosomes could be confused with other particles that may have similar characteristics, such as some lipoproteins. It is therefore important to complement these methods with the detection of exosomal protein markers to support the accurate isolation of these vesicles.

#### 8.2.3. Detection of Proteic Exosomal Markers

The International Society for Extracellular Vesicles (ISEV) has presented extensive recommendations for marker-based exosome characterization [324], some of which include the detection of transmembrane proteins common to all exosomes, such as tetraspanins (i.e., CD63, CD9 or CD81) as well as integrins, selectins and CD40 ligands [324]. As many of these proteins are involved in normal physiology and disease pathogenesis, they are used as important pathophysiological biomarkers of extracellular vesicles [325]. Other exosomal markers include intraluminal proteins, such as TSG101, ALIX, annexins and Rabs. It is also possible to detect any protein unique to exosomes in order to differentiate from lipoproteins that may have been co-isolated [315]. Characterization of these protein markers can be performed by Western blot, Enzyme-Linked ImmunoSorbent Assay (ELISA) or flow cytometry [315,325].

Western blot is an easy and widely known procedure. It is characterized by its accessibility and ability to detect exosomal surface and internal proteins. However, its specificity and reproducibility are limited by the quality of the antibody used, and a large amount of exosomal protein is usually needed to obtain a good signal regarding a few proteins each time [2,325]. Like Western blot, ELISA can analyze marker proteins qualitatively and quantitatively and its limit of detection is similar. However, processing is faster, making it suitable for high-throughput analysis [325]. On the other hand, flow cytometry allows for the size measurement and the observation of the structure of exosomes; while conventional flow cytometers can only measure particles larger than 300 nm, thus not allowing direct detection of exosomes [289], the new generation of flow cytometers are provided with an improved resolution, enabling the quantification and/or classification the exosomes according to the level of antigen expression detected by fluorescent antibodies or stains that will emit a signal detected by the machine [323]. Their challenge is focused on avoiding aggregation (clumping) of vesicles, which would produce inaccurate results in exosomal numbers and/or sizes [314]. In some cases, immobilization of exosomes on a bead surface is sometimes preferred, either by immunocapture or by covalent conjugation. Then, the exosomal vesicles are exposed to a fluorescently conjugated antibody against an antigen known or expected to be expressed on the exosomal surface [2,314].

### 8.3. Methods for the Study of Exosomal Content

Analysis of the content of exosomes provides information on the differential expression of their biomolecules. This could be valuable for the discovery of potential biomarkers for early diagnosis and prognosis of disease, as well as for monitoring patient response to treatment [2]. Methodological aspects for the study of exosomal proteins, RNA and lipids will be briefly reviewed.

Exosomal proteins have been analyzed by different proteomic strategies in various cell types and biofluids to investigate whether they can be used as potential biomarkers for diagnosis [289]. For this type of analysis, the sample must first be prepared to separate, extract, and digest the exosomal proteins [316]. There are two common methods for this aim: in-gel digestion (sodium dodecyl sulfate polyacrylamide gel electrophoresis–SDS-PAGE) and in-solution digestion (cold acetone, trichloroacetic acid or methanol/chloroform precipitation methods). Compared to in-solution digestion, in-gel digestion has an advantage, where contaminants, such as detergents, lipids and polymeric materials that are used in different exosome isolation protocols, and could affect further result, can be removed from the samples during electrophoresis [289]. The generated peptides are then fractionated by liquid chromatography. The selection of a particular proteomic approach depends on the research objective. Complete identification of exosomal protein cargo can be achieved with a “global discovery” approach using multidimensional fractionation strategies followed by high-resolution mass spectrometric (MS) [326], which could include a quantitative cargo analysis if desired. This quantification can be performed with the Stable Isotope Labeling by Amino acids in Cell culture (SILAC) or Isobaric Tag for Relative and Absolute Quantitation (iTRAQ) methods, involving the incorporation of a stable heavy isotope-labeled amino acid into the peptides of interests [327,328]. However, these are expensive protocols, and the peptide of interest is not always known. Therefore, label-free techniques have been developed to quantify identified proteins based on spectral counting, i.e., the number of times a specific peptide is selected for fragmentation in a MS [2]. The use of this global discovery approach in the field of extracellular vesicles, and especially exosomes, has been of great utility for the discovery of biomarkers in different diseases.

Another approach is the targeted proteomic analysis, in which only specific proteins are detected and quantified [329]. The most common method is the multiplexed multiple reaction monitoring (MRM), which is often used as a method for validation of biomarkers detected in MS [330].

A targeted method has a lower detection limit and a higher dynamic range compared to global discovery methods. However, the former is the method of choice to identify and quantify, with high reproducibility, preselected peptides in a complex mixture [329]. This preselection of exosomal proteins can be carried out with the following considerations: to include proteins that are involved in specific cellular pathways, proteins that are predicted to be affected under a certain condition or treatment prior to exosome isolation, or proteins detected as altered in the global proteomic analysis [316].

Like proteins, and to identify exosomal RNA as a potential circulating biomarker, this biomolecule has been investigated in various pathologies [331], including cancer [332], inflammation [333] and neurodegenerative diseases [333,334]. Conventional cellular nucleic acid extraction tools, such as phenol-chloroform extraction and centrifuge column techniques, have been successfully used for the particular case of exosomal RNA. The phenol-based method is based on phase separation by centrifugation. The nucleic acid is split into the aqueous phase, which is later recovered by ethanol precipitation. This approach is arduous and time-consuming but could potentially provide RNA of higher purity [315]. Spin columns, on the other hand, relies on the strong binding of nucleic acids to silica in the presence of chaotropic agents [335]. Both extraction approaches have been developed and commercialized with different names and degrees of success [336].

For the study of this biomolecule, next-generation sequencing (NGS) techniques have been used to map all exosomal RNAs. This analysis generates millions of reads with good depth and coverage to facilitate the discovery and characterization of the entire transcriptome [336]. Thus, NGS has made significant contributions to the understanding of exosomal RNA content and distribution, providing great information on the molecular mechanisms mediated by exosomes, useful for biomarker discovery. The results obtained by NGS are commonly validated with real-time polymerase chain reaction and immunoassays, which include the use of commercial antibodies against miRNA [289].

The study of exosomal lipids, on the other hand, starts with vesicle lysis and lipid extraction, which are performed in a single step by liquid-liquid phase extraction [289,337], using, for instance, an organic solvent system (4:1 tetrahydrofuran:water mixture), followed by the separation of phospholipids and glycosphospholipids using diethyl ether and water. Another alternative is to perform the extraction with a Bligh-Dyer-based method, where most types of lipids are dissolved in the organic layer of a chloroform/methanol/water (1:1:1:1) solution [338]. These lipids can be analyzed by gas chromatography-mass spectrometry [339], liquid chromatography-mass spectrometry or by direct infusion electrospray ionization in a high-resolution mass spectrometer [340,341].

## *9.* Methods Involved in Exosome-Based Drug Delivery Systems

In recent years, exosomes have shown promise as drug delivery carriers, providing new opportunities for the treatment of various diseases. Thus, various methodologies related to drug loading and drug release have been proposed.

### 9.1. Methods Involved in Therapeutic Exosomal Loading (Cargo)

Approaches to incorporate cargoes into exosomes can be divided into two: (i) loading before exosome isolation and (ii) loading after exosome isolation. In the first case, therapeutic molecules can be incorporated into the cells of origin before they produce extracellular vesicles, allowing encapsulation of said molecules at the time of exosome biogenesis. Cell transfection of RNA, propeptides and therapeutic proteins have been used for this purpose [342,343].

Another technique used in the pre-isolation loading approach involves exposing cells to the drug molecule without genetic manipulation or modification, where drugs are encapsulated into the exosomes during their biogenesis by passive diffusion [344].

Post-isolation loading methods, on the other hand, aim to incorporate the drug after the exosome collection and isolation process. These include direct co-incubation, sonication, electroporation, freeze-thaw cycles and extrusion [345,346].

Direct co-incubation is a simple method that involves mixing the drug with the purified exosomes and incubating the mixture at room temperature. Since no additional mechanical or electrical force is used, the loading efficiency is relatively lower when compared to other methods, and is tremendously dependent on the hydrophobicity of the drug [347]. Thus, it has been shown that hydrophobic molecules are loaded more efficiently into exosomes by this method than hydrophilic ones. Another factor to consider is the molecular size of the drug since diffusion is inversely proportional to molecular size [315].

During sonication, energy and mechanical force are applied to cause the temporary formation of pores in the exosomal membranes [346]. It is possible that this procedure reorganizes the lipid bilayer, thus facilitating drug penetration into the exosomes [346]. It has been shown that after one hour of incubation the exosomal lipid bilayers recover their initial structure, which is a crucial step in retaining the contents of exosomes [348]. Compared to direct incubation, sonication has a relatively higher loading efficiency. However, this technique could lead to deformation of exosomes by altering their integrity [315].

Electroporation changes the dielectric state of the cell membrane by means of electrical pulses, thereby increasing the permeability and allowing the entry of the cargo [315]. This method has a high loading efficiency. However, aggregation of therapeutic nucleic acids during encapsulation is a limitation [349].

Fast freeze-thaw cycles have also been proposed as a post-exosomal isolation drug loading method. It consists of freezing the drug and exosomes in liquid nitrogen or −80 °C and thawing at room temperature for about three cycles [315]. This results in a slight disruption of the lipid bilayer of the exosomes allowing the drug molecules to penetrate. This method has a moderate loading efficiency, which is lower than the efficiency achieved by the sonication method [350].

The extrusion technique consists of subjecting the exosomes to a process of decomposition of their structure into free lipid and protein molecules. This is achieved using filters with specific pore sizes at controlled temperatures and pressures. Subsequently, the vesicle components are reassembled giving rise to a new population of exosome-like nanoparticles. This process is done in the presence of the drug of interest to integrate it into the new vesicle [351]. The loading efficiency of this technique is higher than that of electroporation. However, this method has the disadvantage that it can induce changes in the protein composition of the vesicle and its drug-release activity [315].

To ensure that the molecule has been efficiently loaded into the exosomes, two methods have been proposed: direct and indirect. In the former, lysis of the exosomes and a drug extraction process are necessary to measure the amount incorporated drug. In contrast, indirect calculation methods measure the amount of unincorporated drug, so that the loading efficiency can be estimated by subtracting the concentration of free drug from the total amount.

### 9.2. Methods Related to Drug Release

Once the drug has been loaded into exosomes, the challenge is to achieve tissue-specific release of the therapeutic molecule, thereby reducing toxicity and side effects while maximizing its efficacy.

As mentioned in Section 4 of this article, exosomes can be internalized by cells in a specific manner, thanks to the recognition of exosomal surface molecules by a target cell receptor [352]. In this sense, these receptor-ligand interactions can be exploited for the targeted release of drugs loaded into exosomes. Thus, genetic and chemical methods have been developed in order to express ligands on the exosomal surface that would allow them to be recognized in vivo by the receptors on the target cells [353].

These strategies include genetic engineering and chemical modification [345]. Genetic engineering fuses the genetic sequence of a guide protein or polypeptide with that of a selected exosomal membrane protein. These sequences are then transfected into a cell that will encode them, and then secrete exosomes with these surface proteins expressed in their surface [353]. This approach is highly effective since it introduces ligands that will direct the exosome to a specific tissue; however, it is only possible if the motifs to be expressed are genetically encodable, which is not always the case [354].

Chemical modification, on the other hand, allows the insertion of a wide range of ligands, both natural and synthetic, through conjugation reactions or lipid assembly [353]. Conjugation reactions can covalently and stably modify the amine groups of exosome surface proteins with alkyne groups. They can now be conjugated with azide-containing motifs through “click chemistry” reactions [355]. This allows conjugation of small molecules and macromolecules that would act as ligands on target cells. This method requires a short time to be completed, which is clearly advantageous, but may affect vesicle structure and function [353].

Lipids or amphipathic molecules can also be inserted into the lipid bilayer of exosomes, allowing their hydrophilic parts to be displayed on the outside [356]. But it should be noted that this method, driven by lipid self-assembly, may increase the toxicity of exosomes [353].

All these drug loading and release techniques are highly dependent on the quality, purity and characterization of the isolated exosomes, as well as the optimal conditions of their storage until use [357]; therefore, future studies are needed to control for these factors and thus ensure that an adequate therapeutic dose of exosomes is delivered to the target tissue.

The size of the extracellular vesicles could influence loading efficiency and the release kinetics of the therapeutic agents that they transport to target cells. Haney et al. [358] evaluated the catalase-loading capacity of exosomes using various methods: incubation at room temperature (RT) with or without saponin, freeze/thaw cycles, sonication and extrusion. Incubation of catalase with exosomes at RT (with or without saponin) did not significantly alter the size of the exosomes. However, the freeze/thaw, extrusion and sonication cycles did cause a significant change of size. In general, the diameter of the catalase-loaded exosomes was in the range of 100 to 200 nm, with the sonicated exosomes being the largest. Despite this, exosomes were absorbed at levels considerably higher than those subjected to freeze/thaw cycles, or incubation at RT. It is speculated that a rearrangement of exosomes after sonication may result in the exposure of hydrophobic parts of the cellular lipid bilayers, or may cause proteins to enhance their interactions with the plasma membrane of the target cells. Other methods, such as extrusion and saponin permeabilization also produced high-loading efficiency, sustained release and conservation of catalase against degradation of proteases [358].

Nevertheless, it is still not clear whether size can influence the loading capacity of exosomes. Hence, more research is required to elucidate the importance of exosome size, and how its change might impact the use of exosomes as disease markers or new therapeutic agent.

## 10. Conclusions and Perspectives

The secretion of exosomes from various types of cells, their existence in almost all types of body fluids and their participation in cell-cell communication at short and long distances, modulating the activity of the receptor cell, give exosomes an important role in physiological and pathological processes. Therefore, exosomes can be measured in different biological fluids and used as potential diagnostic biomarkers.

In this review we have summarized recent findings and potential uses of exosomes and their cargo in the diagnosis of various diseases. We also discussed the rationale for the main methods for exosome detection and isolation. The selection of the ideal technique for use in the clinical setting is a major challenge due to the different advantages and disadvantages of each method. In addition, a better understanding of the biological functions, as well as verification of the sensitivity and specificity of each biomarker under well-defined conditions, is needed before conclusions can be drawn based solely on their concentrations in biofluids.

Finally, due to the stability and packaging properties of exosomes, these vesicles may acquire a central role in the future treatment of complex diseases. Compared to traditional vectors for gene and drug therapy, such as viruses, polyethylenimine nanoparticles and liposomes, exosomes show greater advantages in terms of therapeutic effect, capacity to cross biological barriers, targeting accuracy, low immune response and safety, making exosomes an extraordinary vehicle for biological drugs (e.g., nucleic acids, enzymes, etc.) or small molecules that could potentially have disease-modifying effects.

Furthermore, the potential therapeutic role of MSC-derived exosomes to treat several degenerative diseases has been highlighted within a cell-free exosome-based therapy, since these vesicles, compared to their stem cells, are more stable and can reduce the inherent safety risks in administration, such as the risk of occlusion in the microvasculature. Nevertheless, clinical research has not yet taken off, and an important number of clinical studies are going to be needed in order to guarantee their efficacy. The lack of such studies may be due, at least in part to the fact it has been challenging to ensure a high yield of purified exosomes. Increasing the yield, at least for now, requires an enormous amount of cells culture reagents, space and equipment that is of course translated into higher costs of production. Also, there is a lot of room to improve and optimize the necessary conditions for the maintenance of the exosome-secreting cells, as well as the standard operating procedures, that could improve the reproducibility of results between studies. Thus, it is necessary to increase the understanding of the biology of these nanovesicles to overcome the technical and practical challenges that limit their clinical use today. Currently, our research group studies the differential expression of ncRNA in subjects with Alzheimer’s disease compared to healthy subjects. We also plan to study the exosomal load in patients with attention deficit–hyperactivity disorder [359,360,361,362,363,364] and HIV and Huntington’s disease [365,366].

## Figures and Tables

**Table 1:** Exosomal molecular contents.

Molecules	Examples	Functions	References
cytosolic proteins	clathrin	exosome biogenesis	[40]
	HSC70, HSP70, HSP60, HSP90	exosome secretion and signaling	
	ALIX, YWHAE, ubiquitin, TSG101	exosome biogenesis	
cell surface proteins	CD63, CD9, CD81, CD37, CD68, CD82, LAMP-2B	exosome biogenesis	[40]
	MHCI, MHCII	presentation of antigens to generate immune response	
membrane-associated proteins	annexin I, II, IV, V, VII; RAB7, RAB11, RAB1B	biogenesis and exosome secretion	[53]
cytoskeletal proteins	tubulin, actin, cofilin, profilin I, elongation factor 1a, fibronectin	biogenesis and exosome secretion	[40]
enzymes	ATPase, pyruvate kinase, fatty acid synthase	metabolism	[40]
lipids	cholesterol	exosome secretion	[40,56]
	gangliosides	exosomal rigidity	
	ceramides	classification of exosome loading and exosome secretion	
	phosphatidylserines	exosome formation, signaling and uptake	
	phosphatidylethanolamines, phosphatidylcholines, phosphatidylinositols	exosome formation and structure	
	glycosphingolipid, sphingomyelins	rigidity and signaling	
nucleic acids	mRNA, miRNA, noncoding RNA, mtDNA	protein translation, gene regulation in host cells	[57]
metabolites	carboxylic acids, carnitines, biogenic amines, vitamins, cyclic alcohols.	metabolic products, homeostasis	[54]

**Table 2:** Recent studies describing the potential role of exosomes as biomarkers for various diseases.

Diseases		Exosome Molecule Studied	References
neurodegenerative diseases	Alzheimer’s disease	Aβ-oligomer, p-tau	[199]
		miR-135a	[200]
		BACE1-AS	[201]
		miR-34b, miR-125b and miR -130b	[202]
		GAP43, neurogranin, SNAP25, and synaptotagmin 1	[203]
		hemoglobin	[204]
	Parkinson’s disease	α-Synuclein and clusterin	[205]
		α-Synuclein, STX-1A and VAMP-2	[206]
		let-7d, miR-15b, miR-24, miR-142-3p, miR-181c, and miR-222	[202]
		miR-153 and miR-223	[207]
cancer	breast	CD9, CD44 and EpCAM	[190]
		miR-7641	[188]
		combination of miR-1246 + miR-206 + miR-24 + miR-373	[189]
	cervix	miR-125a-5p	[191]
		CircEIF4G2	[192]
	prostate	miR-423-3p	[195]
		miR-532-5p	[193]
		miR-375, miR-451a, miR-486-3p andmiR-486-5p	[194]
	lung	miR-1268b and miR-6075	[195]
		miR-1246 and miR-96	[196]
		linc01125	[197]
complications during pregnancy	preeclampsia	miRNA-153, miRNA-325, miRNA-342-3p miRNA-653-5p, miRNA-222-3p, miRNA-224-5p and miRNA-532-5p	[208]
	gestational diabetes mellitus	miRNA-125b and miRNA-144	[209]
		miR-516-5p, miR-517-3p, miR-518-5p, miR-222-3p and miR-16-5p	[210]
cardiovascular diseases	acute myocardial infarction	hsa-miR-1180-3p, hsa-miR-3615, hsa-let-7i-5p, hsa-miR-106b-5p, hsa-miR -143-3p, hsa-miR-17-5p and hsa-miR-1273h-3p; 1	[211]
		miR-126, miR-21 and PTEN	[212]
		lncRNA: ENST00000556899.1 and ENST00000575985.	[213]
	unstable angina pectoris	miR-126, miR-21 and PTEN	[212]
infectious diseases	COVID-19	fibrinogen alpha, beta and gamma chains, fibronectin, complement subcomponent C1r and serum amyloid P component	[175]
		CD235a+, CD14+, CD8+, CD19+, CD4+, CD19+ and CD146+ exosomes	[214]
		CRP, alpha-1-acid glycoprotein 1 and 2, chemokine ligand 7, zinc-alpha-2-glycoprotein, coiled-coil domain-containing protein 34, and complement component 4 binding protein alpha	[175]

**Table 3:** Recent studies describing the potential role of exosomes in the treatment of disease.

Diseases		Exosome Molecule Studied	References
Neurodegenerative diseases	Alzheimer’s disease	neprilysin	[247]
		quercetin	[248]
		miR-21, miR-29b y miR-146a	[249]
	Parkinson’s disease	miR-188-3p	[250]
		miR-7	[251]
		miR-30a-5p	[252]
	Other neurological disorders	miR-21, miR-193b y miR-216a	[249]
Cancer	Transport of chemotherapeutics	Paclitaxel	[243]
		Cisplatin	[244]
		Doxorubicin	[246]
	Cancer immunotherapy	M1 macrophage-derived exosomes.	[253]
		CAR-T cell-derived exosomes	[254]
	Biological reprogrammers of cancer cells	miR-139-5p	[255]
		miR-381	[256]
		miR-140-3p	[257]
		miR-5100	[258]
		miR-1249, miR-126, miR-27b, 520a, miR-590-5p and miR-622	[259]
Cardiovascular diseases	Cellular conditioning	miARN-21-5p	[260]
		miR-146a, miR-181b y miR-126	[261]
		βARKct-CDC exosomes	[262]
Infectious diseases	Bacterial infections	antimicrobial peptides: cathelicidin LL-37, human β-defensin-2 (hBD-2), hepcidin and lipocalin-2 (Lcn2).	[263]
	Sepsis	miR-27b	[264]
		miR-21	[265]
		super-repressor IκB	[266]
	COVID-19	CD24 and T cell-derived exosomes	[267,268,269,270]
		MSC-derived exosomes (ExoFlo^®^)	[271]
		Zofin^TM^	[272]

## Data Availability

No new data were created or analyzed in this study. Data sharing is not applicable to this article.
